# Multi-Year Regional-Scale Efficacy Assessment of Two Herbicides for Treatment of Invasive Floating Aquatic Vegetation in a California Estuary

**DOI:** 10.1007/s00267-026-02625-8

**Published:** 2026-09-23

**Authors:** Shruti Khanna, Jereme W. Gaeta, Krista Hoffmann, Susan L. Ustin

**Affiliations:** 1 Interagency Ecological Program, Bay Delta Region 3, California Department of Fish and Wildlife, Stockton, CA USA; 2 University of California, Davis, CA USA; 3 Water Branch, California Department of Fish and Wildlife, Sacramento, USA; 4 Wildlife Branch, California Department of Fish and Wildlife, Sacramento, USA

**Keywords:** Glyphosate, 2, 4-D, Remote sensing, Management, *Pontederia crassipes*, *Ludwigia*

## Abstract

Invasive aquatic macrophytes are a major threat to estuarine ecosystems globally. This study examined the efficacy of a program for treating invasive Floating Aquatic Vegetation (FAV) using two herbicides, glyphosate and 2,4- Dichlorophenoxyacetic acid (2,4-D), over two five-year periods. The efficacy of both these herbicides has rarely been studied in tidal aquatic environments that are much more challenging to treat. This study combined multi-year image datasets, spray location and herbicide application data to examine the efficacy of the treatment program at the scale of the full ecosystem in a tidally dynamic estuary. The objectives of the study were 1) to determine if the herbicides type, application rate and frequency had an impact on efficacy, 2) if efficacy depended on the kind of habitat that was treated and the treatment history of the site, and finally, 3) if the impact lasted years after treatment. The results show that within the year of application, probability of FAV presence reduced as herbicide application rate increased. Both herbicide treatments were most effective in slow-flow shallow open-water habitats, and least effective in fast-flowing channel habitats. Treating with glyphosate had legacy effects that were detectable even three years after treatment but only in some habitats. For either herbicide, treating at a higher concentration in fewer visits was more effective than treating at a lower concentration over multiple visits to the same site.

## Introduction

From driving ecosystem regime shifts to native species extinctions, invasive species have a range of harmful effects and are a leading driver of biodiversity loss in aquatic ecosystems (van Donk and van de Bund 2002; Wilson et al. 2007; Molnar et al. 2008). Invasive species can severely affect ecosystem processes such as nutrient cycling and hydrology (Gordon 1998; O’Farrell et al. 2009) leading to shifts between alternative stable ecosystem states (Scheffer and Jeppesen 2007; O’Farrell et al. 2009; Pejchar and Mooney 2009). Invasives can also hinder human uses of aquatic systems such as recreation and water transport (Charles and Dukes 2007; Pejchar and Mooney 2009; Jetter and Nes 2018). Invasive macrophytes impact aquatic communities including invertebrate distribution, diversity, and abundance (Meerhoff et al. 2003; Toft et al. 2003; Stiers et al. 2011; Vanderstukken et al. 2011), increase anoxic conditions detrimental to fish and other aquatic life (Dandelot et al. 2005, 2008; Wilson et al. 2007; Nehring and Kolthoff 2011), and inhibit fish movement (Stiers et al. 2011; Thouvenot et al. 2013). The competitive ability of invasive macrophytes, for example, water primrose (Dandelot et al. 2008; Drexler et al. 2024), may come from the ability to secrete allelopathic chemicals that reduce germination and seedling survival, or by reducing light availability for other species, for example, water primrose and water hyacinth (Malik 2007; Wilson et al. 2007; Stiers et al. 2011), while being able to take advantage of low-light conditions, for example, alligator weed (Longstreth and Mason 1984; Geng et al. 2007). However, managing and controlling aquatic non-native macrophytes has proven an extremely difficult challenge in many systems (Hussner et al. 2017).

In the United States, vast resources are spent on controlling the spread of invasives (Pimentel et al. 2005) and on preventing or mitigating the impact of these species (Lovell et al. 2006). Water hyacinth (*Pontederia crassipes* (Mart.) Solms, formerly, *Eichhornia crassipes*; www.usda.gov, www.usgs.gov), native to Brazil (Penfound and Earle 1948), and water primrose (*Ludwigia* spp.), native to South America (Sears et al. 2006), are two highly invasive Floating Aquatic Vegetation (FAV) species that have invaded multiple continents (Gopal 1987; Thouvenot et al. 2013). Many strategies for controlling FAV have been proposed starting with manual removal for incipient species, mechanical removal in early stages of invasion, herbicide treatment once the species is well established, and biocontrol for long-term maintenance (Malik 2007; Thiebaut 2007; Thouvenot et al. 2013). However, each of these methods have limitations. Manual and mechanical control can be prohibitively costly and costs rise steeply with limited accessibility (Penfound and Earle 1948; Meisler 2009). Additionally, *Ludwigia* spp., for example, can regrow from small fragments, and mechanical control can create and spread such fragments leading to dispersal rather than control (Rejmánková 1992; Nehring and Kolthoff 2011). Biocontrol of FAV is insufficient for heavy infestations and thickly growing mats (Malik 2007; Thouvenot et al. 2013; Hopper et al. 2017). Invasive species management programs that use both biocontrol and chemical control methods can sometimes suppress the biocontrol agent impeding the success of biocontrol (Hill et al. 2012). For example, herbicides and the surfactants used in applying them, both led to significant mortality of weevils and mirids used for biocontrol of water hyacinth in South Africa (Hill et al. 2012).

Several herbicides have been used for treating FAV (Penfound and Earle 1948; Malik 2007; Thouvenot et al. 2013; Sartain et al. 2015), however, the two with the longest historical use are glyphosate and 2,4-D (Hildebrand 1946; Eggler 1953; Gutierrez et al. 1996). 2,4- Dichlorophenoxyacetic acid (2,4-D) was patented for herbicide use in 1945 by Franklin D Jones (Peterson 1967) and is a systemic herbicide effective on broadleaf dicot species without affecting monocots. 2,4-D acts by altering the plasticity of the cell walls, influencing the amount of protein production, and increasing ethylene production. This leads to uncontrolled growth causing stem curl-over, leaf withering and eventual plant death (Song 2014). Glyphosate (N-(phosphonomethyl)glycine) was patented by Monsanto in 1974 for herbicide use. It is a broad-spectrum systemic herbicide that targets the enzyme, 5-enolpyruvyl-shikimate-3-phosphate synthase (EPSPS) (Duke and Powles 2008). Glyphosate is taken up rapidly by leaves but not by stems or roots, and has good translocation capability (Duke and Powles 2008). Even though glyphosate was developed almost three decades after 2,4-D, it became more commonly used due to the development of glyphosate resistant crops and its early reputation as a safe herbicide (Duke and Powles 2008). However, there is lack of consensus on harmful impacts of glyphosate with some studies indicating that it is not toxic to animals and humans as it binds to soil leaving little residue in water or groundwater (Giesy et al. 2000), while others indicate that glyphosate might be carcinogenic at higher doses and that the surfactants used with glyphosate make the herbicide mixture toxic at much lower dosage levels (Gill et al. 2018; Martins-Gomes et al. 2022). 2,4-D is known to be even more toxic to non-target organisms such as zooplankton, fish, humans, and crops (de Castro Marcato et al. 2017).

Studies of efficacy focused on glyphosate and 2,4-D for FAV control in aquatic habitats have often relied on mesocosm experiments (Eggler 1953; Robles et al. 2010; Sartain et al. 2015; Riner et al. 2025). Some studies have looked at efficacy directly in the field (Gutierrez et al. 1996; Meisler 2009; Madsen and Kyser 2020; Kyser et al. 2021), but with only a few sites and over a few weeks with the exception of Meisler (2009). Aquatic environments are harder to monitor and robustly test for efficacy, because, in contrast to a terrestrial site, you require a specialized vessel like a flat-bottom boat or an airboat, which involves specialized safety training and significantly more “man-hours” just to reach the sample site (Madsen and Wersal 2017). Remote sensing offers an alternate low-cost option to monitor efficacy of herbicide treatment in aquatic habitats. Multiple studies have assessed the potential of Unmanned Aerial Vehicles (UAV) and satellite imagery to track herbicide impact on FAV species (Robles et al. 2010; Sartain et al. 2019; Yousef et al. 2019; Riner et al. 2025). However, very few image-based studies span several years or a large spatial scale (Santos et al. 2009; Yousef et al. 2019). Santos et al. (2009) were pioneers in using aerial spectroscopy-derived vegetation maps to study treatment efficacy of floating and submerged invasive aquatic vegetation in the Sacramento-San Joaquin Delta (henceforth, Delta) but they only had access to the first five years of data from this long-term dataset (Khanna et al. 2025).

The state of California spends more than 13 M USD a year to treat invasive aquatic plant species in the Delta (Conrad et al. 2022). The objective of our study was to evaluate the annual treatment program in the Delta at the landscape scale over multiple years. The large spatial and temporal scale of this study allowed us to examine efficacy of the program from multiple aspects. We test the following hypotheses across two time periods, 349 sites from 2004 to 2008 and 333 sites from 2014 to 2018:

1. Herbicide specifications: a) type: is glyphosate or 2,4-D more effective in treating FAV; b) application rate: does the total amount of herbicide sprayed per unit area during a treatment season affect efficacy; c) frequency: do the number of visits to a site over a treatment season affect herbicide efficacy since sites are often visited multiple times in a single treatment season?
2. Site specifications: a) site history: do consecutive years of treatment at the same site have an impact on efficacy of the herbicide; and b) habitat type: Does the impact of treatment differ across sites with different hydrologic characteristics from more lentic to more lotic?
3. Long-term impact: a) does the treatment history of a site (how many years it was treated consecutively) affect the durability of treatment impact; and b) how long does the impact last after a treatment season?

Answers to these questions have direct management implications for the control program in the Delta, and more broadly, for informing science-based planning for landscape-scale treatment programs for FAV in tidal aquatic environments.

## Data and Methods

### Study Area

**Fig. 1:**
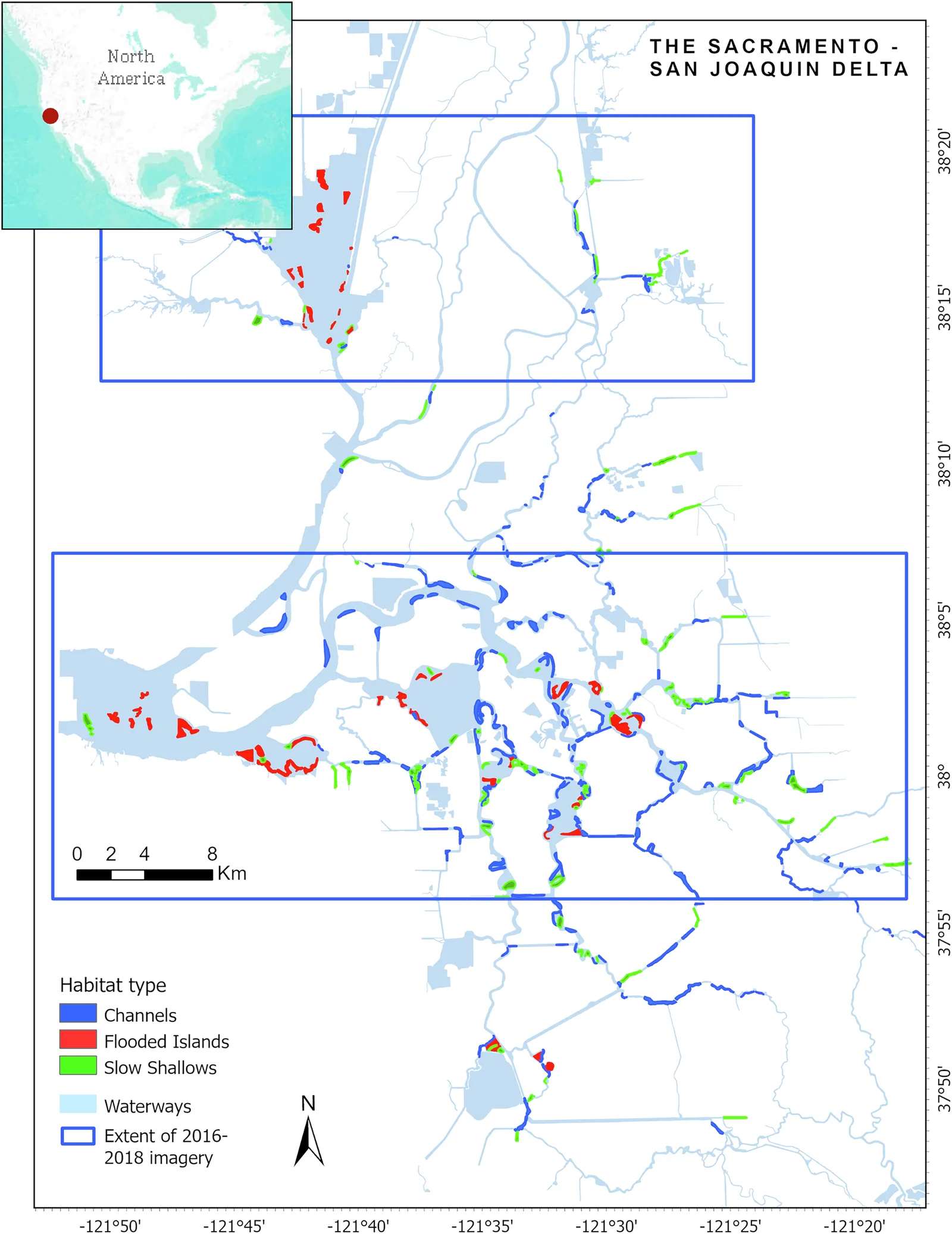
Location of the Sacramento – San Joaquin Delta in the United States (inset) and all treatment and control sites included in the study categorized by habitat type. Blue boxes show the extent of the imagery available for the years 2016–2018. Only the sites present within the blue boxes were included in the study for those years

The Delta, spread over 2220 km^2^ in northern California, is an inverted Delta formed by the draining of two major watersheds in northern and central California, the Sacramento River watershed and the San Joaquin River watershed (Fig. 1). Before the mid-19th century, the Delta was comprised of extensive freshwater tidal marshes with few open-water environments (Atwater et al. 1979). Since then, over 90% of the tidal marshes have been diked (Atwater et al. 1979), water control infrastructure have been built, and water has been rerouted into straighter paths (Nichols et al. 1986). Hence, the modern Delta retains a few remnant tidal marshes within meandering channels separated by shallow interconnecting expanses of water (slow shallows), inundated lake-like islands that have arisen from sporadic levee failures (flooded islands), and channels with shallow banks but relatively faster moving water within the channel. These three habitats have different hydrology that may influence the efficacy of treatment for FAV.

The two main non-native FAV species infesting the Delta are water hyacinth and water primrose (mainly *Ludwigia hexapetala*). In the past couple of decades, two more alien FAV have been detected in the Delta, spongeplant (*Limnobium laevigatum*) in 2008 and alligator weed (*Alternanthera philoxeroides*) in 2017 (Christman et al. 2022). While three of the FAV species are truly floating, water primrose is a creeping emergent – it roots in the substrate at the water edge or at the bottom of the water column and sends creepers out on the water’s surface (Rejmánková 1992). However, water primrose has specialized roots sticking out of the water and adventitious roots in the water to access nutrients in the water column (Sears et al. 2006), thus behaving similar to FAV although it might be harder to dislodge. Water hyacinth has been found in the Delta since 1904 and water primrose since 1949 (Light et al. 2005), but the dominance of water primrose has increased post-2014 (Khanna et al. 2018; Christman et al. 2022). Both these species have had major negative impacts on the Delta ecosystem (Khanna et al. 2012, 2018; Christman et al. 2022), the local economy, and the water conveyance infrastructure (Khanna et al. 2019).

### The FAV Treatment Program

The extensive treatment program for managing FAV in the Delta began after a 1982 legislation (California State Senate, Senate Bill 1344, Regular session 1982) which funded the treatment program (Conrad et al. 2022). The California State Parks, Division of Boating and Waterways (DBW) is the primary entity responsible for managing this program initially called the Water Hyacinth Control Program (WHCP), eventually renamed to the Aquatic Invasive Plant Control Program (AIPCP) in 2019 (Carruthers et al. 2012; Ta et al. 2017; Conrad et al. 2022). From 1983 to 2000, the main herbicide used for treating FAV was 2,4-D, however, as concerns have grown over its impact on adjacent agricultural crops and endangered fish species, its use has been slowly phased out. In the early years of the treatment program, since 2001, 2,4-D was the main herbicide, but over the last two decades, glyphosate has been used with increasing frequency (Conrad et al. 2022). Since 2014, systemic herbicides imazamox and penoxsulam have been used on a very limited basis to diversify the herbicide portfolio, aiming to reduce the risk of target plants developing resistance to the dominant herbicides (Conrad et al. 2022). However, the use of these new herbicides is not widespread enough to include them in this remote sensing based comparative study. This study straddles the switch from 2,4-D to glyphosate (2009–2013) as the primary herbicide used for treating FAV in the Delta (see Online Resource 1: Fig. 1). The AIPCP uses 2,4-D dimethylamine salt at the rate of 1.7 to 3.4 kg∙ha^−1^ and glyphosate isopropylamine salt at the rate of 4.6 kg∙ha^−1^ of active ingredient (Caudill et al. 2021). The adjuvant used is Agridex. The time between re-applications also differs between 2,4-D and glyphosate. While glyphosate can be sprayed 24 h later, 2,4-D needs 21 days between reapplications (Carruthers et al. 2012). The herbicides are applied as a foliar spray.

The scale and longevity of the FAV treatment program in the Delta allows an evaluation of treatment efficacy at regional and multi-year scales. In addition to a long-running treatment program in this system, high-quality genus-level maps have been produced for FAV species in the Delta for two time periods (2004–2008 and 2014–2025) from hyperspectral imagery (Khanna et al. 2025). The availability of detailed treatment records in the same timeframe make possible a landscape-scale evaluation of treatment impacts, the legacy effects of those impacts after treatment ceases, and how efficacy may vary among the two herbicides contingent on treatment history and hydrologic habitat.

### Data and Workflow

Vegetation class maps derived from aerial spectroscopy data were used to calculate the response variable per site. The target classes from the maps (e.g. water, submerged aquatic vegetation, water hyacinth, water primrose, etc.) were reclassed to FAV/non-FAV and the pixel counts of each class summarized for each site. For detailed information on image data and preprocessing steps, see Online Resource 1 and the data publication (Khanna et al. 2025).

Covariates were derived from a dataset that integrated four distinct datasets. A detailed description of source data used is presented in Online Resource 1. Herbicide application log tables, boat path vectors, and vegetation class maps derived from image data were integrated to compute the following information for each site treated in any year during the study period:

1. Herbicide type: 2,4-D or glyphosate
2. Application rate per unit area for the entire treatment season (ml/m^2^)
3. Frequency of spraying within a treatment season (sprFreq)
4. Consecutive treatment years until the present year (CY). A value of CY = 0 in any year indicates that this site was untreated for that year and hence a reference site.

The consecutive treatment years were further consolidated into a categorical variable (CY2yrs) to ensure enough sample size within each category to allow for a robust model. The relationship between CY and CY2yrs is shown in Table 1.

**Table 1:** Conversion from continuous years of treatment (CY) to CY2yrs

CY	CY2yrs	Description
0	0	Not treated in current year (Reference sites)
1, 2	1	Treated for 1–2 consecutive years
3, 4	2	Treated for 3–4 consecutive years
5, 6	3	Treated for 5–6 consecutive years
7	4	Treated consecutively for >6 years

Additionally, to explore the legacy effects of the herbicide on FAV occurrence, we computed two covariates from the treatment data:

1. For all sites that were not treated in the current year but treated the previous year, we calculated the continuous treatment years for the site until the previous year (CY_t-1_) and the corresponding CY2yrs_t-1_. The relationship between CY_t-1_ and CY2yrs_t-1_ was the same as described in Table 1 between CY and CY2yrs.
2. For all sites not treated in the current year, we computed a treatment flag (T_flag_) which is defined as the number of years since the site was last treated. We only considered sites that were treated 1, 2 or 3 years prior to current year. Less than 10% of the sites were treated more than 3 years ago.

The hydrologic characteristics of a site can also affect the efficacy of the herbicide. The Delta supports a wide variety of habitats, but it can be broadly characterized into three hydrologically distinct habitats: channels that are deep, with high tidal connection, have sharp bathymetry at the banks, and have low water residence times; slow shallows that are moderately tidally connected shallow regions between remnant wetland patches within larger channels; and flooded islands that are lake-like areas with remnant levees surrounding them, low tidal action, and high water residence time (see Online Resource 1: Fig. 2).

**Fig. 2:**
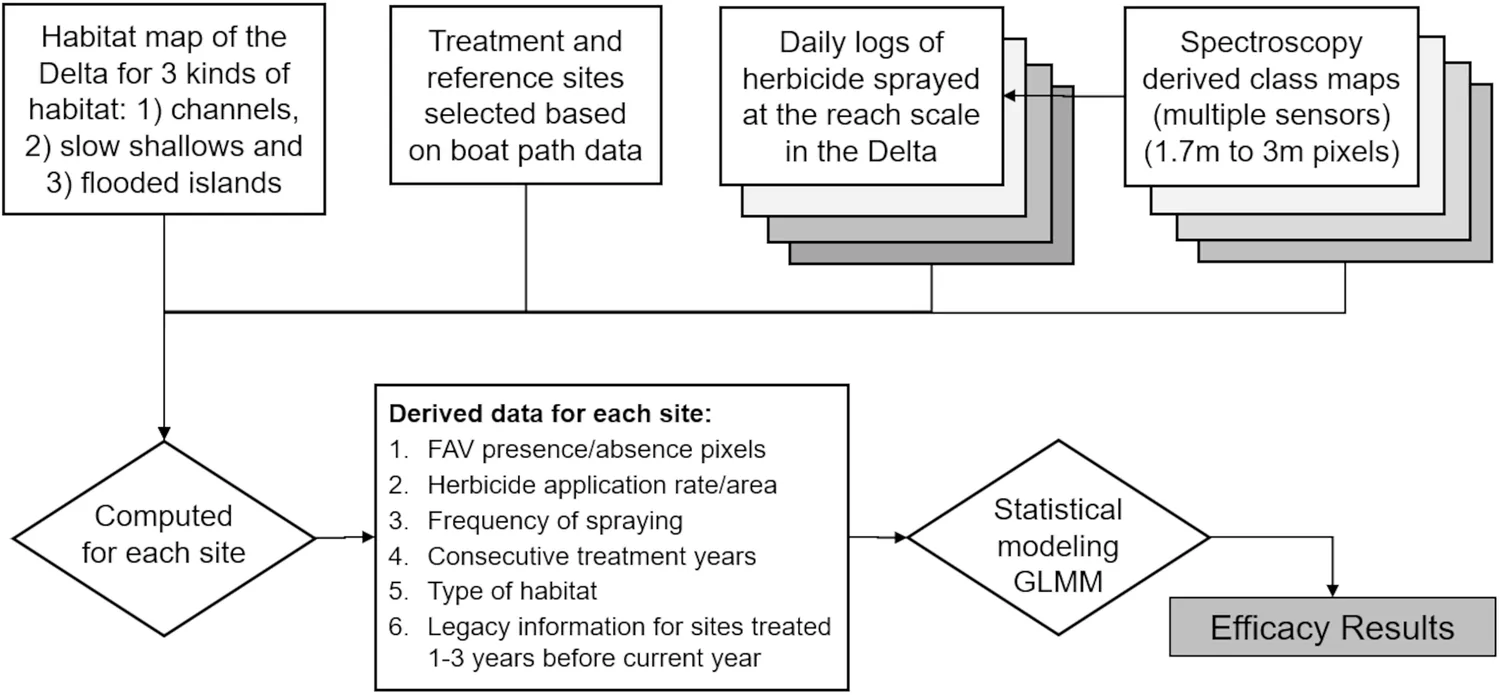
Input data used to derive the integrated dataset and workflow of the study

We explored the role of FAV patch size, hypothesizing that larger patches might be harder to treat than smaller ones. We also considered that frequency of spraying might be related to patch size since bigger patches might require repeat visits. However, we found no relationship between patch size and spray frequency. Additionally, we could not add more covariates beyond the five we were already considering and still have sufficient statistical power to explore all of them. Hence, patch size was not included as a covariate in the final study design.

Input datasets and derived data are summarized in Fig. 2.

We modeled two main scenarios in this study: the effect of treatment on a site within the year of application, and the legacy effect of treatment, 1–3 years after application.

### Treatment Impact Within Year of Application

We evaluated herbicide efficacy using a hypothesis-driven approach in which we tested whether the probability of FAV presence was a function of herbicide applied per unit area, frequency of treatment within a single treatment season, and consecutive years of treatment at a site. We took a longitudinal mixed effects logistic (binomial) regression approach with the non-nested random effects (random intercepts) of site and year using the following model structure:

$${y}_{i} \sim {Binomial}\left({c}_{i},\,{p}_{i}\right)S1$$

$$\begin{array}{c}{p}_{i}\,=\,{{logit}}^{-1}\left({\beta }_{0}\,+\,{\beta }_{1}{x}_{{1}_{{jk}}}+{\beta }_{2}{x}_{{2}_{{jk}}}+{X}_{{jk}}{\beta }_{x}+{\alpha }_{j\left[i\right]}+{\alpha }_{k\left[i\right]}\right),\,{for}\,i\,=\,1,\ldots ,\,n\end{array}$$

$${\alpha }_{j}\, \sim \,{{\rm{N}}}\left(0,\,{\sigma }_{{\alpha }_{j}}^{2}\right),\,{for}\,j\,=\,1,...,\,J$$

$${\alpha }_{k}\, \sim \,{{\rm{N}}}\left(0,\,{\sigma }_{{\alpha }_{k}}^{2}\right),\,{for}\,k\,=\,1,...,\,K$$

Where ${y}_{i}$ is the number of pixels with FAV present given the total pixel count ${c}_{i}$ of the site with the mean probability of ${p}_{i}$ for observation i. Therefore, ${p}_{i}$ is the ${i}^{{th}}$ observation of the proportion of pixels with FAV cover within the ${j}^{{{\rm{th}}}}$ site in the ${k}^{{{\rm{th}}}}$ year and can be interpreted as the probability of FAV presence. ${\beta }_{0}$ is the model intercept; ${\beta }_{1}$ is the coefficient of ${x}_{1}$, ${\log }_{10}{herbicide\; ml}\bullet {m}^{-2}$ (log to base 10 transformed to maximize normality); ${\beta }_{2}$ is the coefficient of ${x}_{2}$, $\root 3 \of {{sprFreq}}$ (cube-root transformed to meet the assumption of normality); ${X}_{{jk}}{\beta }_{x}$ is a matrix of the possible CY2yrs (${X}_{{jk}}$; treated as a categorical variable) and the corresponding suite of coefficients (${\beta }_{x}$); ${\alpha }_{j}$ is the random intercept of site with a mean of 0 and variance of ${\sigma }_{{\alpha }_{j}}^{2}$; and ${\alpha }_{k}$ is the random intercept of year with a variance of ${\sigma }_{{\alpha }_{k}}^{2}$.

The three habitats were modeled separately. The two time periods were also modeled separately (2004–2008 and 2014–2018) resulting in six total models for this part of the study. In the 2004–2008 time period, we included sites that were only treated with 2,4-D and not with glyphosate. In the 2014–2018 time period, we included sites that were only treated with glyphosate and not 2,4-D. Thus, we took advantage of the change in herbicide treatment regime between the two time periods to study the effect of each herbicide independent of the other.

### Legacy Effect of Treatment

Legacy effect or the continuing impact of treatment after treatment has ceased was tested in two ways. First, we tested if continuous years of treating a site strengthened the legacy effect of the herbicide observed more than a year after cessation of treatment. Second, we tested how many years after the herbicide application we could still detect its impact on the site. We used a hypothesis-driven approach to test whether the probability of FAV presence during non-treatment years was a function of continuous years of treatment of the previous year, before treatment stopped (i.e., at time *t-1*) using the CY2yrs to allow for more observations within each category value. Effectively, we evaluated whether multiple years of herbicide treatment would result in reduced probability of FAV presence upon cessation of treatment using a longitudinal, mixed effects logistic (binomial) regression approach with the non-nested random effects (random intercepts) of site and year using the following model structure:

$${y}_{i} \sim {Binomial}\left({c}_{i},\,{p}_{i}\right)S2$$

$$\begin{array}{c}{p}_{i}\,=\,{{logit}}^{-1}\left({\beta }_{0}\,+{X}_{{jk}}{\beta }_{x}+{\alpha }_{j\left[i\right]}+{\alpha }_{k\left[i\right]}\right),\,{for}\,i\,=\,1,...,\,n\,\end{array}$$

$${\alpha }_{j}\, \sim \,{{\rm{N}}}\left(0,\,{\sigma }_{{\alpha }_{j}}^{2}\right),\,{for}\,j\,=\,1,...,\,J$$

$${\alpha }_{k}\, \sim \,{{\rm{N}}}\left(0,\,{\sigma }_{{\alpha }_{k}}^{2}\right),\,{for}\,l\,=\,1,...,\,K$$

where ${y}_{i}$ is the number of pixels with FAV present from observation i given the total observation pixel count ${c}_{i}$ with the mean probability of ${p}_{i}$. ${p}_{i}$, therefore, is the ${i}^{{th}}$ observation of the proportion of pixels with FAV cover within the ${j}^{{{\rm{th}}}}$ site during the ${k}^{{{\rm{th}}}}$ year and can be interpreted as the probability of FAV presence when not treated with herbicide. ${\beta }_{0}$ is the model intercept; ${X}_{{jk}}{\beta }_{x}$ is a matrix of the possible CY2yrs from the previous year (${X}_{{jk}}$; treated as a categorical variable) and the corresponding suite of coefficients (${\beta }_{x}$); ${\alpha }_{j}$ is the random intercept of site with a mean of 0 variance of ${\sigma }_{{\alpha }_{j}}^{2}$; and ${\alpha }_{k}$ is the random intercept of year with a variance of ${\sigma }_{{\alpha }_{k}}^{2}$. The model was fitted to each habitat individually.

We explored how long the legacy effect of treatment can last by fitting a similar model described above but where ${X}_{{jk}}$ is a treatment flag (T_flag_) that is defined as the number of years since a site was last treated. The three habitats were modeled separately for both the formulas described above resulting in six separate models. Since the sites that were included for examining legacy effects were only sites that were not treated in year t, and treated in year t-1 or t-2 or t-3, the sample sizes for studying legacy effects are much smaller than that for studying the effect of herbicide within the year of application. Statistical analyses were performed using R CRAN Statistical software (R Core Team 2020; version 4.0.2) using package ‘lme4’ (version 1.1-2.6).

## Results

**Fig. 3:**
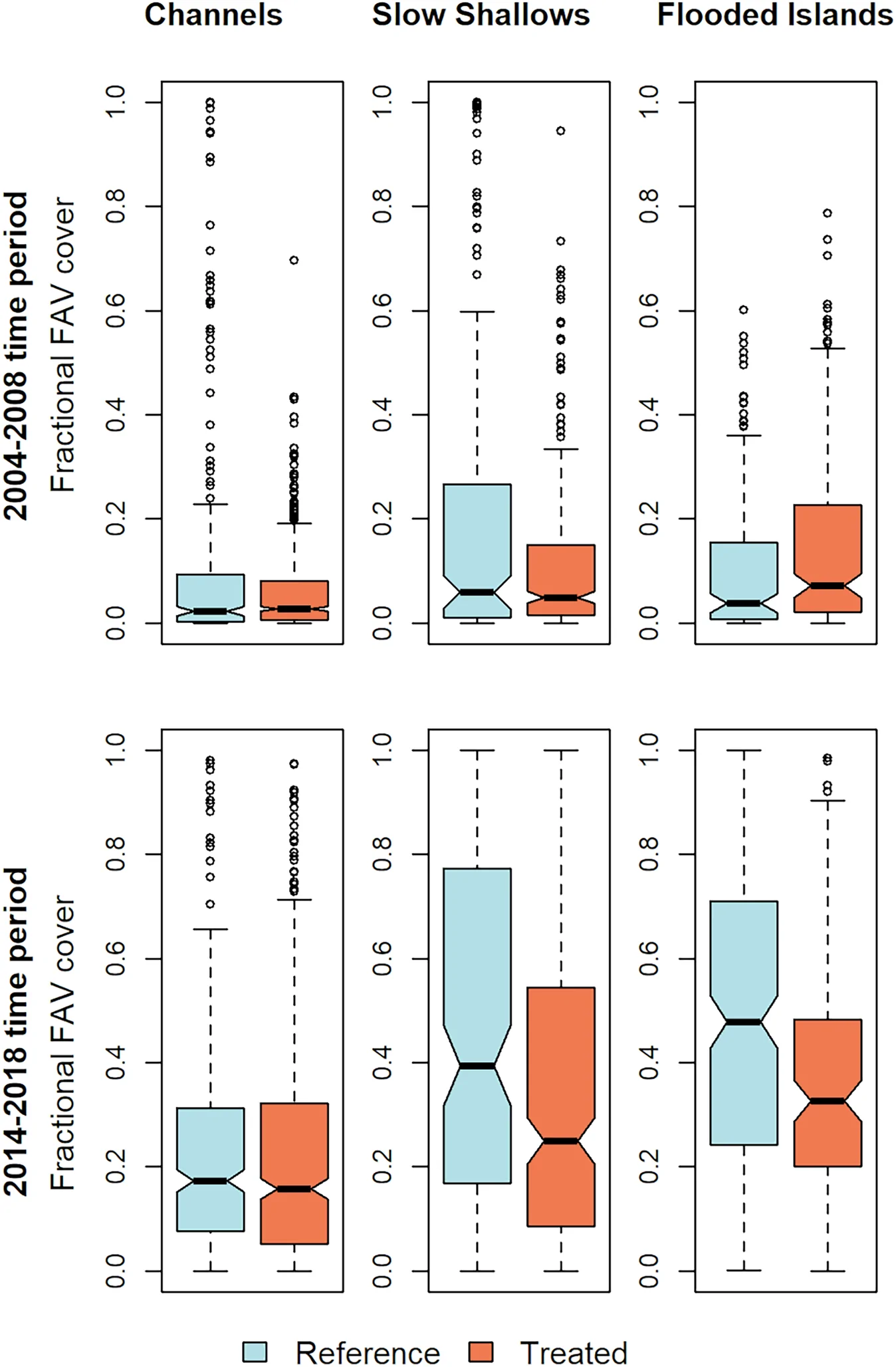
Boxplots of percent cover of FAV in treated and reference sites in all three habitats (columns) and both time periods (rows)

FAV percent cover and the probability of FAV occurrence in 2004–2008 was, on average, lower than that in 2014–2018 across all three habitats (Fig. 3). Across both time periods, the highest percent cover of FAV and probability of FAV occurrence was observed in the slow shallows, then flooded islands, and the lowest probability was observed in channel habitats (compare columns in Fig. 3).

### Treatment Impact Within Year of Application

Model fit for the six models developed for the three habitats and two time periods was assessed following the procedures described in Zuur et al. (2009) and Gelman and Hill (2006) by plotting residuals against covariates. See Online Resource 1: Tables 3–8 for model parameter values and Online Resource 1: Figs. 3–8 for a visual assessment of model fit.

**Fig. 4:**
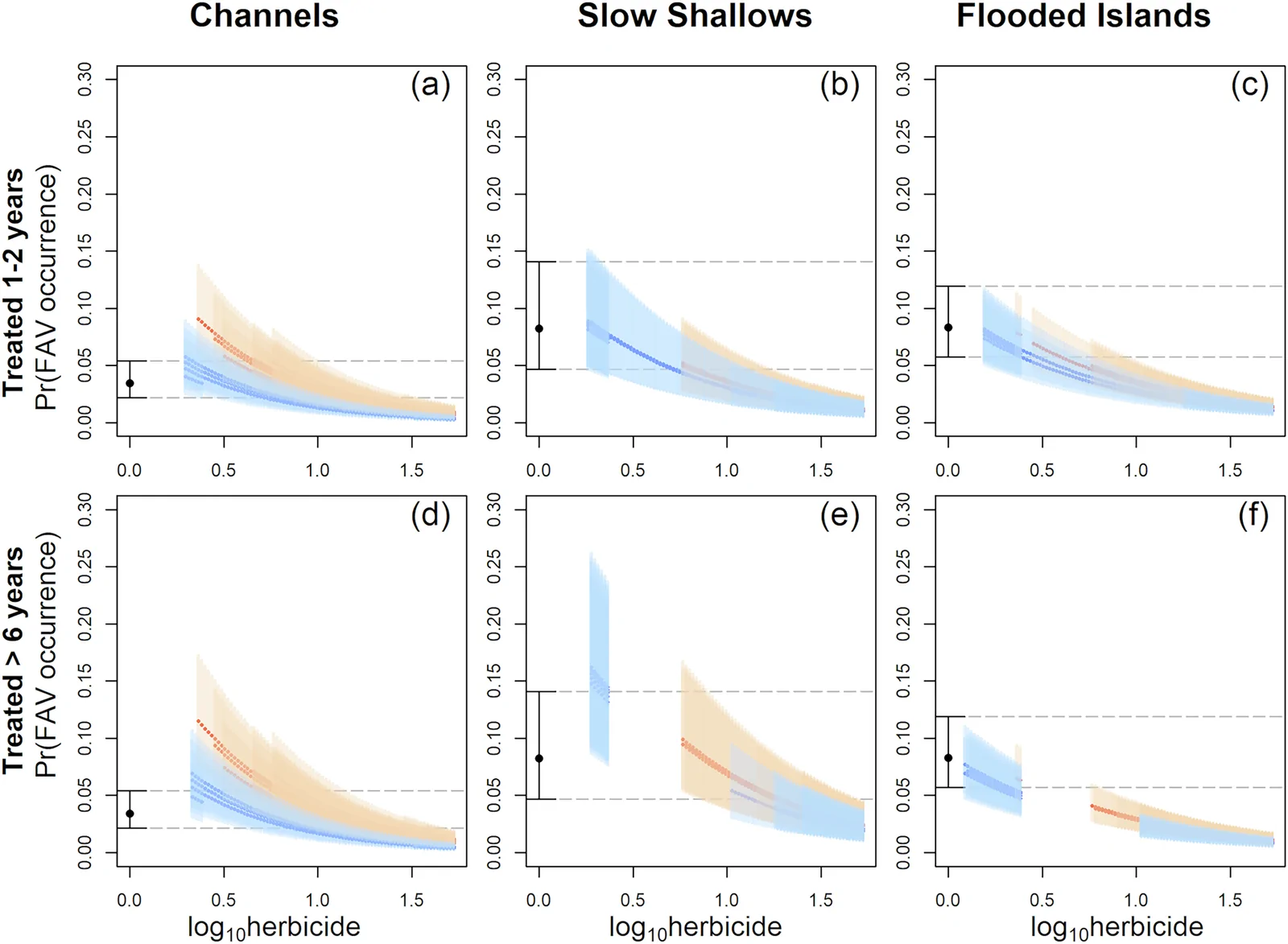
Simulated model results for the 2004–2008 time period testing the efficacy of the 2,4-D herbicide. Top row shows the probability of FAV occurrence in sites only treated for 1–2 years consecutively ﻿(panels **a**, **b** and **c** for each of the three habitats), while the bottom row shows the probability of FAV occurrence in sites treated for more than 6 years consecutively (panels **d**, **e** and **f** for each of the three habitats)﻿. Predicted FAV occurrence values for spray frequencies over 70 percentile are shown in red, while predicted values for spray frequencies less than 30 percentile are shown in blue. Each individual blue or red series corresponds to a single value of spray frequency. Gaps in the plots indicate combinations of spray frequency and herbicide application rate that did not occur in our dataset hence were not simulated. Probability envelopes for reference polygons are shown as the point estimate when no herbicide is sprayed (*x* = 0) and extended with dashed gray lines for easy comparison

To disambiguate the effects of the three covariates included in the models, we took a simulation approach to evaluate our hypotheses. We simulated datasets including 100 values of herbicide application rates constrained between 5 and 95 percentile of its distribution in our dataset, every value of spray frequency, and every value of CY2yrs for each region and each time period. FAV occurrence was predicted using the corresponding model for each simulated dataset using the ‘effects’ package (version 4.2-0). Figure 4 shows the simulated results for the 2004–2008 time period using the 2,4-D herbicide.

#### Effect of herbicide type

**Fig. 5:**
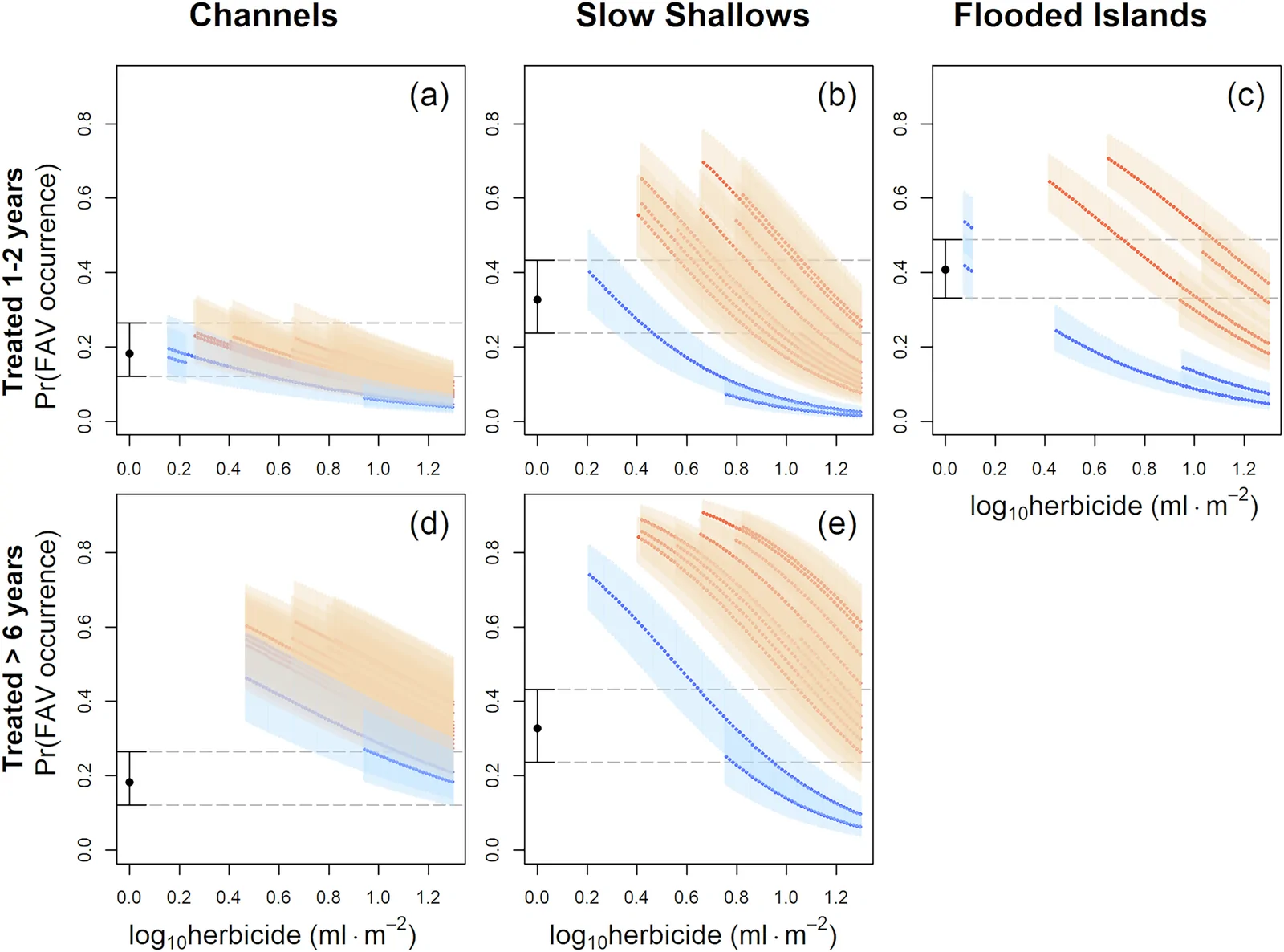
Simulated model results for the 2014–2018 time period testing the efficacy of the herbicide, glyphosate. Top row shows the probability of FAV occurrence vs. herbicide application rate in sites only treated for 1–2 years consecutively (﻿panels **a**, **b** and **c** for each of the three habitats) while the bottom row shows the results in sites treated for more than 6 years consecutively ﻿(panels **d** and **e** for channels and slow shallows respectively; there were too few sample sites to analyze results for flooded islands). Predicted FAV occurrence values for spray frequencies over 70 percentile are shown in red, while predicted values for spray frequencies less than 30 percentile are shown in blue. Each individual blue or red series corresponds to a single value of spray frequency. Gaps in the plots indicate combinations of spray frequency and herbicide application rate that did not occur in our dataset hence were not simulated. Probability envelopes for reference polygons are shown as the point estimate when no herbicide is sprayed (*x* = 0) and extended with dashed gray lines for easy comparison

2,4-D was most effective in flooded islands, but less effective in channels or slow shallows. In the latter habitats, impact was only significant at highest herbicide application rates used in the field (Fig. 4). Glyphosate was ineffective in channels at any application rate but it was effective in both slow shallows and flooded island habitats (Fig. 5).

#### Effect of application rate vs. spray frequency

The probability of FAV occurrence decreased as the total amount of herbicide applied in a treatment season increased (Figs. 4 and 5). However, the number of visits over which the herbicide was delivered was also important. In any one panel, the x-axis indicates the total herbicide sprayed in the season. Series of predicted FAV probability for spray frequencies over 70 percentile are plotted in red, while predicted values for spray frequencies less than 30 percentile are plotted in blue. The intersection of these series curves with a line perpendicular to the x-axis represents the same total herbicide sprayed per unit area (or application rate) over an entire treatment season but over a different number of repeat visits. Fewer visits imply a higher amount of herbicide sprayed per visit since the total herbicide sprayed is the same. Thus, the red curves indicate more repeat visits, but lower amount of herbicide sprayed per visit or a lower dose. Similarly, the blue curves represent a higher dose of herbicide sprayed per visit.

For 2,4-D, spray frequency only affected efficacy in channels (Fig. 4a, d) but was not a driver in slow shallows or flooded islands (Fig. 4b, c, e, f). In channels, a higher dose of herbicide in fewer visits was more effective than a lower dose per visit delivered more frequently. This pattern was clearer in glyphosate use in later years. In all 3 habitats, spraying a higher dose of herbicide per visit was more effective than spraying a lower dose of herbicide, even if the total herbicide sprayed per season was the same (Fig. 5). In slow shallows and flooded islands in particular, delivering smaller doses of glyphosate more frequently through the spray season was ineffective in significantly reducing probability of FAV occurrence relative to reference sites.

#### Effect of treatment history

Finally, treatment history or the continuous years of treatment seemed to make no difference in efficacy for 2,4-D (compare top and bottom rows of Fig. 4). In the 2014–2018 time period, we did not have enough of a sample size in flooded islands of sites treated for multiple consecutive years, so we only show the plots for sites treated for 1–2 years consecutively. However, in both channels and slow shallows, sites that were treated with glyphosate for many years consecutively seem to require a higher rate of herbicide application to achieve significant reduction in the probability of FAV occurrence (Fig. 5, a, b compared to d, e).

### Legacy Effect of Treatment

**Fig. 6:**
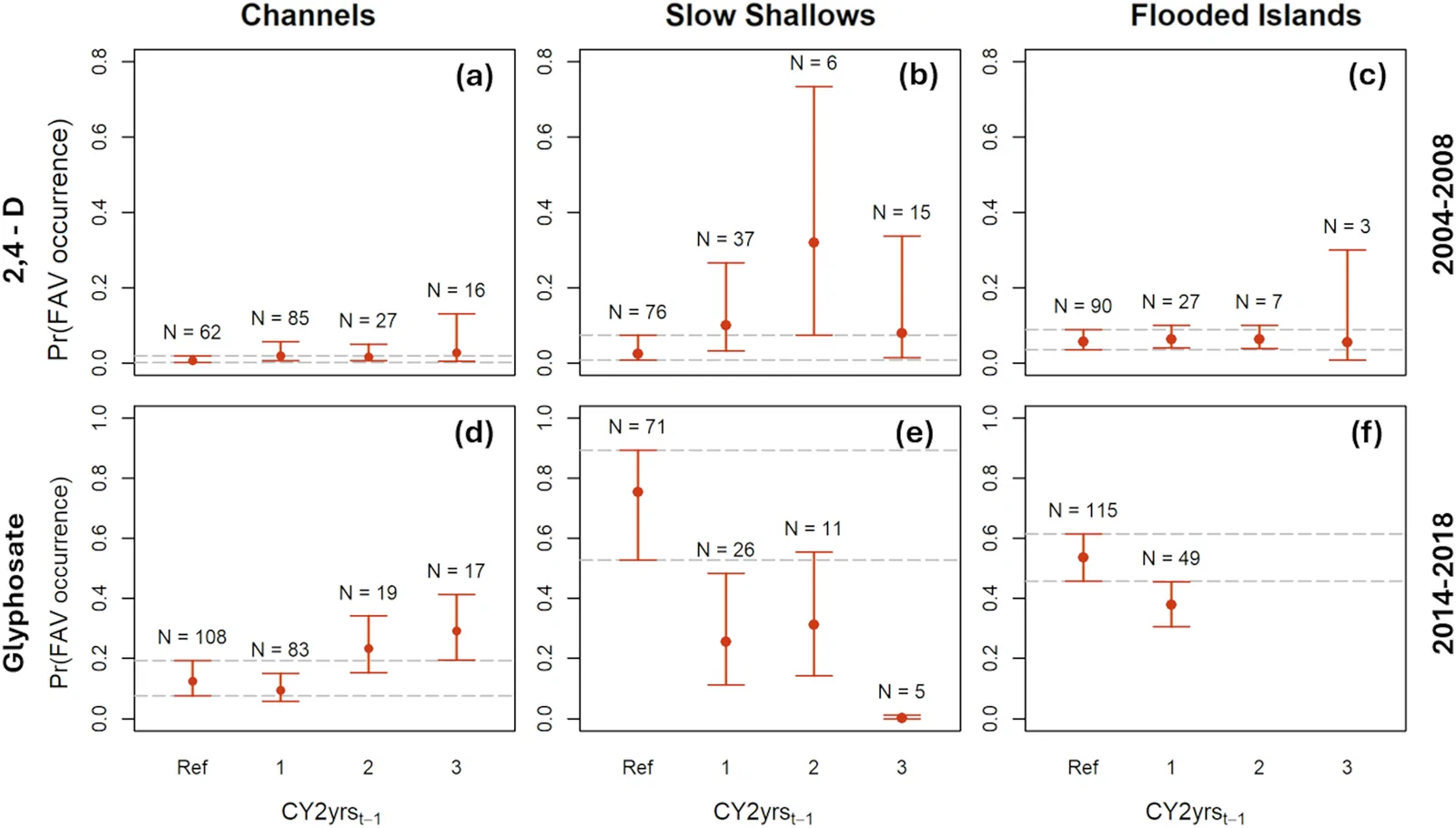
Legacy effects one year after treatment by 2,4-D (top row, panels **a**, **b**, and **c** for channel, slow shallows, and flooded island habitats respectively) and by glyphosate (bottom row, panels **d**, **e**, and **f** for the three habitats) in the corresponding time periods. Plots show the probability of FAV occurrence with the 95% confidence interval in sites not treated in the current year but treated in the previous year also accounting for treatment history until the previous year. These sites are compared to probability estimates for reference sites that have never been treated (gray dashed lines extended for easy comparison). For the relationship between CY2yrs_t-1_ and CY_t-1_, see Table 1. N above the point estimates show the number of samples within each category of CY2yrs_t-1_

Figure 6 shows the legacy effect of *treatment history* of a site, one year after treatment. Online Resource 1: Table 9 shows the parameters and z-values for the 2004–2008 time period and the 2,4-D herbicide, while Online Resource 1: Table 10 shows the parameters and z-values for the 2014–2018 time period and the glyphosate herbicide. Online Resource 1: Figures 9 and 10 illustrate visual assessment of model fit for each corresponding time period.

There are clear differences between the legacy effects of 2,4-D and glyphosate. In channel habitats, neither herbicide is effective in reducing probability of FAV occurrence beyond the year of treatment. In fact, it appears that more consecutive years of treatment may be counter productive to the duration of impact. In slow shallows and flooded islands, 2,4-D still shows no effect or negative effect of higher consecutive years of treatment. However, in slow shallows, glyphosate has more of a lasting impact when sites are treated for 5–6 years consecutively compared with sites treated for less than 5 years consecutively (Fig. 6e). In flooded islands, we did not have many sites that had a long treatment history so we could not study the legacy effect of treatment history beyond two consecutive years of treatment.

**Fig. 7:**
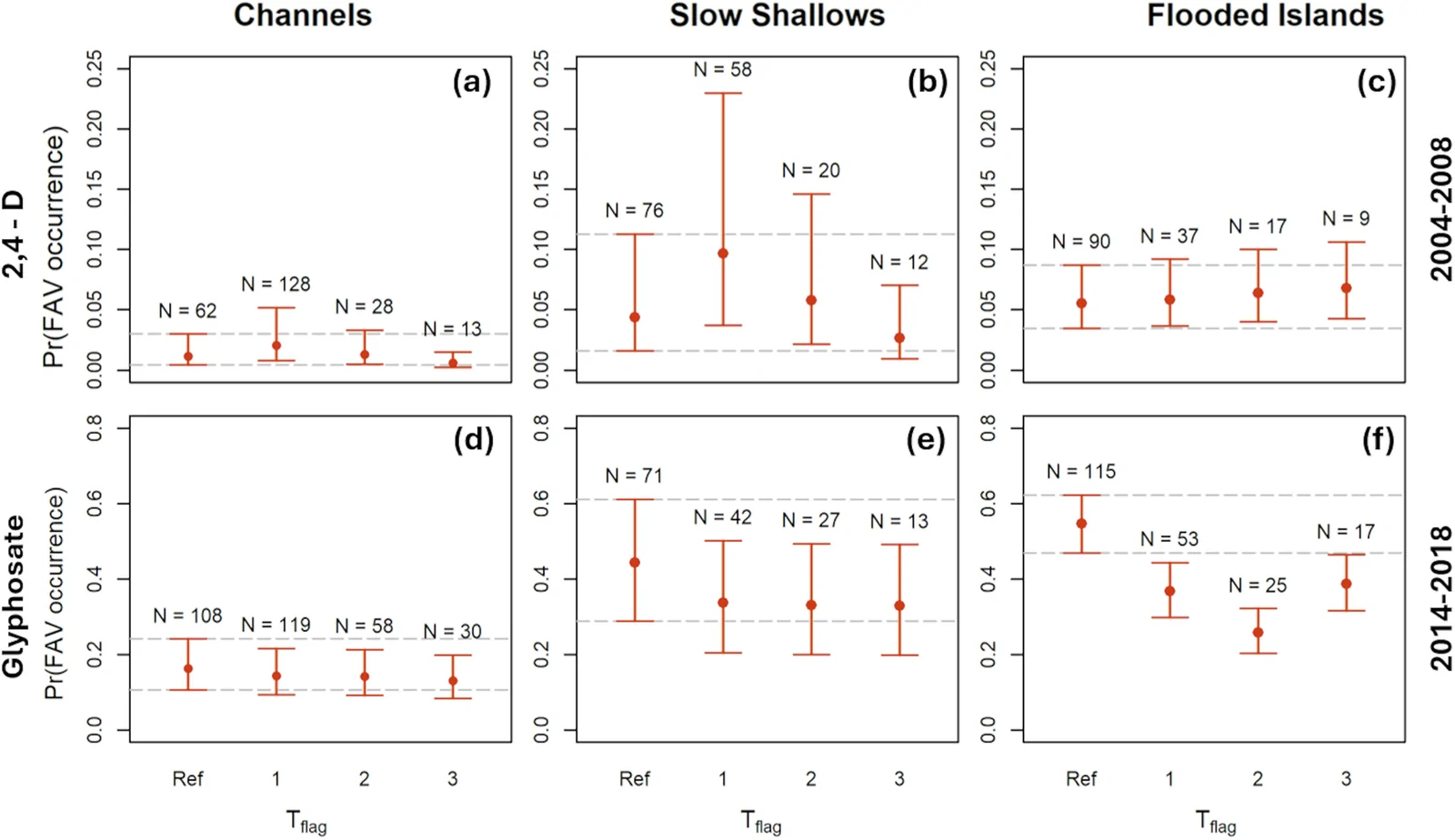
Legacy effects 1, 2 and 3 years after treatment when treated by 2,4-D (top row, panels **a**, **b**, and **c** for channel, slow shallows, and flooded island habitats respectively) and by glyphosate (bottom row, panels **d**, **e**, and **f** for the three habitats) in the corresponding time periods. Plots show the probability of FAV occurrence with the 95% confidence interval in sites not treated in the current year but treated 1, 2 or 3 years before the current year (T_flag_). These sites are compared to probability estimates for reference sites that have not been treated for more than 4 years (gray dashed lines extended for easy comparison). N above the point estimates show the number of samples within each category of T_flag_

Figure 7 shows the legacy effect of each herbicide, 1, 2 or 3 years after treatment. Confidence intervals lower than the gray dashed lines (reference site estimates) indicate that sites treated a year ago still show significant reduction in probability of FAV occurrence. Online Resource 1: Table 11 shows the parameters and z-values for the 2004–2008 time period and the 2,4-D herbicide, while Online Resource 1: Table 12 shows the parameters and z-values for the 2014–2018 time period and the glyphosate herbicide. Online Resource 1: Figures 11 and 12 illustrate visual assessment of model fit for each corresponding time period.

The results for legacy effects using T_flag_ as a covariate are somewhat different than with CY2yrs_t-1_. For 2,4-D, the results show that the effect of treatment is undistinguishable, a year after it ends, regardless of the habitat. For glyphosate, the only sites that show a legacy effect of treatment are in flooded island habitats. In fact, in flooded islands, the probability of FAV cover was significantly lower compared to reference sites, even 3 years after treatment. However, no significant differences were detected in the other two habitats.

## Discussion

Our findings show that the efficacy of herbicide treatments for controlling FAV is a complex interaction between habitat type, spray application rate and frequency. Treatments were generally only effective in slower moving waterways and were more effective at higher herbicide spray rates over fewer visits. Glyphosate exhibited longer lasting results compared to 2,4-D, with reduced FAV presence documented up to three years after glyphosate treatment in some habitats.

### Effect of Habitat

We found that the percent cover of FAV and the probability of FAV occurrence increased from the 2004–2008 to the 2014–2018 monitoring periods across the Delta (see y-axis values between Figs. 3–5). Previous studies have found that propagule pressure is high in more lotic channel environments (Heidbüchel et al. 2020; Thomaz 2025) but seedling recruitment and or establishment of new growth through plant fragments is more successful in lentic environments such as flooded islands (Triest et al. 2010). Miskella and Madsen (2021) conducted a study in water hyacinth mats in the Delta with GPS markers and showed that water hyacinth mats move around frequently and in all directions because of the tidal nature of the Delta. Thus, propagules are constantly traveling and relocating with the movement of water in tidally active regions. Slow shallows have lower flows compared to channels but are more tidally open compared to flooded islands. Hence, it is not surprising that slow shallows have the highest probability of FAV occurrence, likely due to a combination of a nutrient rich environment coupled with the continuous introduction of seeds or plant fragments that are able to take root because of the slow-moving shallow conditions.

Across the treatment periods, FAV treatment efficacy was greater in slow-moving water (e.g. flooded islands and slow shallows) than in fast-moving channels which is consistent with treatment of submersed aquatic vegetation (Khanna et al. 2023), but may be due to different factors. In the faster moving water of channels, propagule pressure from floating mats of FAV in the vicinity (Miskella and Madsen 2021) is likely the major factor responsible for poor treatment efficacy, though water quality conditions and nutrient supply in the channel environment may also promote FAV regrowth (Wilson et al. 2005; Vedder et al. 2021). Flooded islands, on the other hand, are more isolated from the tidal flows carrying floating mats of FAV, therefore treatment effects are more likely to be detectable months and years after application.

### Effect of Spray Rate and Frequency

While reduced herbicide rates are sometimes recommended as a sound practice for environmentally friendly invasive plant control (Lim 2022); research has found that low-dose herbicide applications may be ineffective and can cause long term resistance in weed populations (Gressel 1995; Busi and Powles 2009; Manalil et al. 2011; Norsworthy et al. 2012). Results from our study reinforce the findings that a minimum concentration threshold per application would be necessary for effective FAV control. Low-dose applications of 2,4-D and glyphosate were ineffective in reducing the probability of FAV occurrence relative to reference sites in all habitats evaluated (Figs. 4 and 5). On the other hand, higher application rates of both 2,4-D and glyphosate effectively reduced the probability of FAV occurrence in slow shallows and flooded island habitats when compared to untreated reference sites (shown as blue curves compared to reference values in Figs. 4 and 5).

Treatments were most effective at the highest herbicide rates over fewer visits compared to treatments that occurred across a greater number of visits (demonstrated by the difference between the blue and red curves in Figs. 4 and 5). One factor that may contribute to this effect, but that was not directly evaluated in this study, is the mortality rate (time to lethality). In a separate study, Eggler (1953), found that a relatively high 2,4-D use rate of 9 kg/ha caused the treated water hyacinth mat to sink faster than that treated with a lower concentration, which increased the efficacy of control. At lower application rates, regrowth started before the mat sank and recovery from treatment was possible (Eggler 1953). Notably, treatments of larger patches of FAV are often sectioned out into a patchwork of applications that occur over multiple visits to avoid causing a crash in dissolved oxygen levels from large quantities of decaying matter all at once (Greenfield et al. 2007; Gettys et al. 2009). Initial statistics, however, found no correlation between patch size and number of visits (R^2^ = 0.01); therefore, higher frequency of visits is not skewed towards larger patch sizes that are generally more difficult to control.

In some areas, low dose herbicide applications were not only ineffective, but resulted in a higher probability of FAV presence relative to reference sites (Figs. 4 and 5, the curves fall above the reference values for probability of FAV presence at lower herbicide concentrations). This paradoxical response may be due to post-treatment environmental conditions in established FAV patches. FAV decomposition can alter the aquatic microenvironment by increasing nutrients and reducing light, ultimately favoring FAV recurrence over algal and SAV growth. Another factor at play could be the phenomenon of “hormesis,” which is low-level stress exposure that results in stimulation of cellular growth. While not directly evaluated in this study, this phenomenon is well-documented in toxicant and stress exposure studies (Cedergreen et al. 2007; Belz and Duke 2014). This type of response is due to an adaptive strategy, driven by an organism’s ability to activate and enhance its internal cellular defense and repair mechanisms, and has been documented in plants exposed to a variety of low-level chemical stressors, including 2,4-D and glyphosate (Forsyth et al. 1997; Belgers et al. 2007; Jalal et al. 2021). This is a growing field of research and herbicide hormesis is even being exploited in agriculture to boost crop yield (Duke et al. 2025). In broadscale aquatic applications, hormesis may result from sublethal treatment rates due to purposeful application of lower spray rates, spray drift, or uneven application. Other studies have found that exposure to sublethal concentrations could contribute to a post-treatment population surge (Cedergreen et al. 2007; Belgers et al. 2007). Chinnusamy et al. (2012) demonstrated such a rebound effect in their tank culture experiment, where water hyacinth initially succumbed to 2,4-D applications within 14 days after treatment, but regrowth developed one week later. Notably, glyphosate was tested during the same study, and a rebound effect did not occur in those treatments, presumably because the rate applied was potent enough to cause complete mortality (refer to discussion section below regarding herbicide type). Relating our findings to the explanatory factors found significant in other studies may help to explain the higher probability of FAV occurrence following low-dose 2,4-D and glyphosate treatments, however, further study is needed to confirm that hormesis is occurring.

The lack of efficacy at sites with low-dose 2,4-D and glyphosate applications may also signal the development of herbicide resistance (Ofosu et al. 2023). Treatment over many years with the same herbicide, particularly at low concentrations, can facilitate the development of resistant plants with the effect of hormesis potentially helping to drive this response (Belz et al. 2022). An effect of herbicide resistance seems apparent in our data when comparing effective glyphosate concentrations between Fig. 5a–c that represent sites treated for 1–2 consecutive years, with Fig. 5d, e that represent sites treated for greater than six consecutive years. The data show that a higher concentration is needed to significantly reduce the likelihood of FAV presence relative to reference sites after 6 years of treatment compared to sites only treated for 1–2 years. Herbicide resistance in agricultural settings is quite common (Ofosu et al. 2023), but resistance can also develop in natural plant communities (Richardson 2008). Plant resistance to 2,4-D has been documented across multiple target species and the mechanisms of resistance usually develop from non-target site resistance (NTSR) mechanisms like enhanced metabolism (Grossmann 2010) or reduced translocation (Goggin et al. 2016; Peterson et al. 2016). Glyphosate resistance has also been documented widely in agricultural settings, with similar NTSR documented as well as target site resistance mechanisms relating to a modification in the EPSPS enzyme (glyphosate’s target site of action), allowing it to function even in the presence of herbicide (Palma-Bautista et al. 2023; Zakaria et al. 2025).

### Effect of Herbicide Type and Legacy Effects

Legacy effects were tested using two different models. One model tested if continuous years of treating a site strengthened the legacy effect of the herbicide. The second model tested how many years after the herbicide application we could detect its impact on the site.

While sites treated with 2,4-D over multiple years showed no lasting benefit in all three habitats, glyphosate was effective in reducing the probability of FAV occurrence in both flooded islands and slow shallows but not in channel habitats (Fig. 6). A longer history of treatment was associated with a stronger dampening of FAV occurrence in slow shallows. There were insufficient flooded islands sites with a long treatment history to test this effect in that habitat. Additionally, the second model using T_flag_ as a covariate reinforced that there was no lasting impact of 2,4-D application in all three habitats, one, two or three years after the year of treatment (Fig. 7a–c). Glyphosate similarly showed no lasting impact in channels or slow shallows, however, it did significantly lower the probability of FAV occurrence in flooded islands (Fig. 7d–f). This effect was detectable even three years after the last treatment year (Fig. 7f). The two legacy models, one with CY2yrs_y-1_ as the covariate and one with T_flag_ as the covariate, are built on different sample data. The former only includes sites treated one year ago while the latter includes sites treated 1, 2, or 3 years ago. Hence, they are informing slightly different aspects of herbicide action as described above.

There are two aspects of the study beyond our control that could potentially dampen the effect we observed for 2,4-D impact “within the year of application”. The images in 2004–2008 time period were acquired in July and the herbicide treatment season also began in July. Thus, the imagery is collected right at the start of the spray season. Hence, we used the following year’s imagery to look at the impact of treatment “within the year of application” which was 7–8 months after the treatment season ended. On the other hand, the images in 2014–2018 time period were acquired in fall, just as the treatment season was winding down, hence the impact was being observed directly after treatment instead of months later. Secondly, the probability of FAV cover in general across all three habitats was lower in 2004–2008 compared to later years (Fig. 3). Hence, the impact we are trying to detect is on a smaller scale compared to the latter time period.

## Recommendations for managers

From a management perspective, our results suggest the following recommendations to maximize efficacy and cost-effectiveness of FAV management in the Bay-Delta:

1. Applying a high-concentration dose of herbicide less often is more effective than more frequent sublethal dosing. Sublethal doses should be avoided to prevent stimulating growth (hormesis) or herbicide resistance.
2. FAV patches persist throughout the Delta during the growing season, migrating dynamically with tidal cycles. Herbicide management strategies must account for these dynamics. While channel treatments offer negligible or short-lived efficacy, applications in low-velocity shallows and flooded islands yield more sustained control. Glyphosate’s longer-term efficacy in stable, high-residence time habitats makes it a suitable choice for control in flooded islands, whereas the challenges in channel habitats suggest that neither herbicide may provide the optimal control method.
3. Alternating between herbicide active ingredients may improve efficacy by reducing the likelihood of herbicide resistance (Norsworthy et al. 2012). Post-2018, DBW has begun testing some new herbicide combinations including mixtures of imazamox and penoxsulam along with existing herbicides in smaller Demonstration Investigation Zones (Conrad et al. 2022). These new herbicides require a much lower concentration to be effective, which could improve treatment efficacy over time while being safer for the environment (Madsen and Kyser 2020). Efficacy of these new combinations should continue to be tested in the field.
4. Any weed management program must consider the long-term ecological dynamics within the target area. A holistic management plan should include multiple integrated control methods, active monitoring, and, where feasible, the promotion of native plant communities to prevent re-invasion by the target species or the establishment of new ones (Getsinger et al. 1997; Robles et al. 2011; Khanna et al. 2012; Karouach et al. 2022).

## Supplementary information

Khanna_etal_Suppl_FAVefficacy_forSub

## Data Availability

The datasets used in this study were derived from other datasets. The integrated dataset has been published on the Environmental Data Initiative (https://doi.org/10.6073/pasta/b42537b95445e663549362cd12ac268d).

## Code Availability

All R scripts used to conduct this study are available as a GitHub repository published on Zenodo (https://doi.org/10.5281/zenodo.19056896)
