# Supplementary material for: Multi-Year Regional-Scale Efficacy Assessment of Two Herbicides for Treatment of Invasive Floating Aquatic Vegetation in a California Estuary
Source: Environ Manage. 2026 Sep 23;76(10):326. doi: 10.1007/s00267-026-02625-8 (PMC13601176; doi:10.1007/s00267-026-02625-8)
Supplement: Supplementary file 1 [file 267_2026_2625_MOESM1_ESM.pdf]

Online Resource 1 (Supplementary Material)

Multi-year regional-scale efficacy assessment of two herbicides for treatment of invasive floating aquatic vegetation in a California estuary

Shruti Khanna[1,2], Jereme W. Gaeta[1], Krista Hoffmann[1], and Susan L. Ustin[2]

[1]California Department of Fish and Wildlife, CA, USA; [2]University of California, Davis, CA, USA.

Author for correspondence: Shruti Khanna, California Department of Fish and Wildlife, 2109 Arch Airport Road, Stockton, CA, 95206. (E-mail: shruti.khanna@wildlife.ca.gov; ORCID: https://orcid.org/0000-0003-1724-248X)

Data

Data were integrated from three distinct datasets to test the efficacy of herbicide treatment for floating aquatic vegetation (FAV) in the Delta. The source data and the methods specific to producing these datasets are described in brief in this document, but detailed methods for the image analysis dataset have been published elsewhere and are cited here.

Image data

FAV coverage data came from annual maps produced from hyperspectral imagery acquired in summer of 2004-2008 and Fall of 2014-2018 (Khanna et al., 2022; Ustin et al., 2021). The 2004-2008 imagery was collected using the HyMap sensor (126 bands: 450-2400nm, bandwidth: 10-15nm) operated by HyVista Corp. (New South Wales, Australia) at 3x3m pixel resolution. Imagery in the 2008-2017 period was acquired using the AVIRIS-ng sensor (432 bands: 350-2500 nm, bandwidth: 5-7 nm) at 2.5x2.5m pixel resolution by the Jet Propulsion Laboratory (JPL, Pasadena, California, USA) in November 2014 and September 2015. In October 2016 and November 2017, AVIRIS-ng imagery was acquired over just the northwest and central Delta. The same region was imaged in October 2018 using the HyMap sensor again, but at 1.7x1.7m spatial resolution.

Table 1. Summary of image acquisition from 2004-2018.

Year	Image acquisition	# of Flightlines	Sensor	Pixel Size	Ground truth Data
2004	June 25 – July 7	64	HyMap	3m	2103
2005	June 22 – July 8	64	HyMap	3m	2778
2006	June 21-26	64	HyMap	3m	4164
2007	June 19-21	64	HyMap	3m	2126
2008	June 29 – July 7	48	HyMap	3m	1334
2014	November 14-25	61	AVIRIS	2.5m	1036
2015	September 17-21	61	AVIRIS	2.5m	1375
2016	October 8-9	22	AVIRIS	2.5m	637
2017	November 1	22	AVIRIS	2.5m	891
2018	October 6-9	42	HyMap	1.7m	950

The images were preprocessed to reflectance, geocorrected, transformed and classified using a random forest classifier to produce a location map of the major vegetation communities in the Delta such as submerged macrophytes, FAV species, and emergent marsh (Khanna et al., 2022). Detailed methods for analysis of the imagery are described in the reports published with dataset. Field data collected at the same time as image acquisition was used to train the classifier and to determine the classification accuracy. While the class maps do classify FAV down to the genus or species level, we did not distinguish between genera in our analysis of treatment efficacy.

Herbicide application data

Herbicide application data were derived from two independent datasets:

Boat path vectors: All treatment of invasive species in the Delta is conducted by the Division of Boating and Waterways (DBW), California State Parks. The airboats that DBW crews use for herbicide application log the boat path during travel as vector lines. These vector lines were buffered by 20m to account for the application radius before being provided to the authors for the years 2001-2018. The resulting polygon data made it challenging to determine if a patch was treated or if the boat was merely travelling from one patch location to the next. To ensure accuracy in selection of treatment sites, we chose locations that showed multiple boat paths rather than just a single pass. We demarcated treatment sites by matching patches of FAV mapped in our images with multiple boat paths showing treatment at the patch scale, ensuring similar hydrologic conditions within each treatment site. For comparison, we selected untreated reference patches that had similar hydrologic settings and were in the vicinity of treatment sites and had no boat paths going through them. Sites were selected across the entire range of conditions found in the Delta from slow to fast water speeds, fresh to mildly brackish salinities, and various depths and turbidities. Once the treatment and reference sites were defined, we determined the Consecutive Treatment Years (CY) for each treatment site from 2004 to 2018. For example, if a site in 2014 had already been treated two years in a row (in 2014 and 2013), its CY value was two. A CY value of 1 indicated that the site was treated in the current year but not in the previous year. Note that all sites with CY = 0 in each year were reference sites for that year.

Herbicide application log tables: In their annual reports, DBW crews document date, time, type of herbicide, and amount of herbicide applied at the reach scale. We collected and summarized these reports from 2001-2018. Figure 1 shows how the dominant herbicide sprayed changed from 2,4-D to glyphosate from the first to the second time-period of this study. The entire Delta is divided up into "reaches" for the treatment campaigns run by the agency. These DBW reaches include entire channel sections. Thus, each DBW reach typically has many different patches of FAV and areas without FAV. The daily logs of the amount of each herbicide that was applied within each DBW reach are published annually, but these logs do not have specific GPS locations associated with them. We addressed this uncertainty by integrating the daily logs over the entire treatment season to determine the type and amount of herbicide applied per DBW reach, per treatment season, for each year. Then, within each reach, we integrated the FAV patch area that was treated according to the boat path vectors to calculate the *amount of herbicide applied per unit area of FAV* for all years of study for each reach within the Delta. Each treated site present within a DBW reach was assigned the application per unit area calculated for that reach.

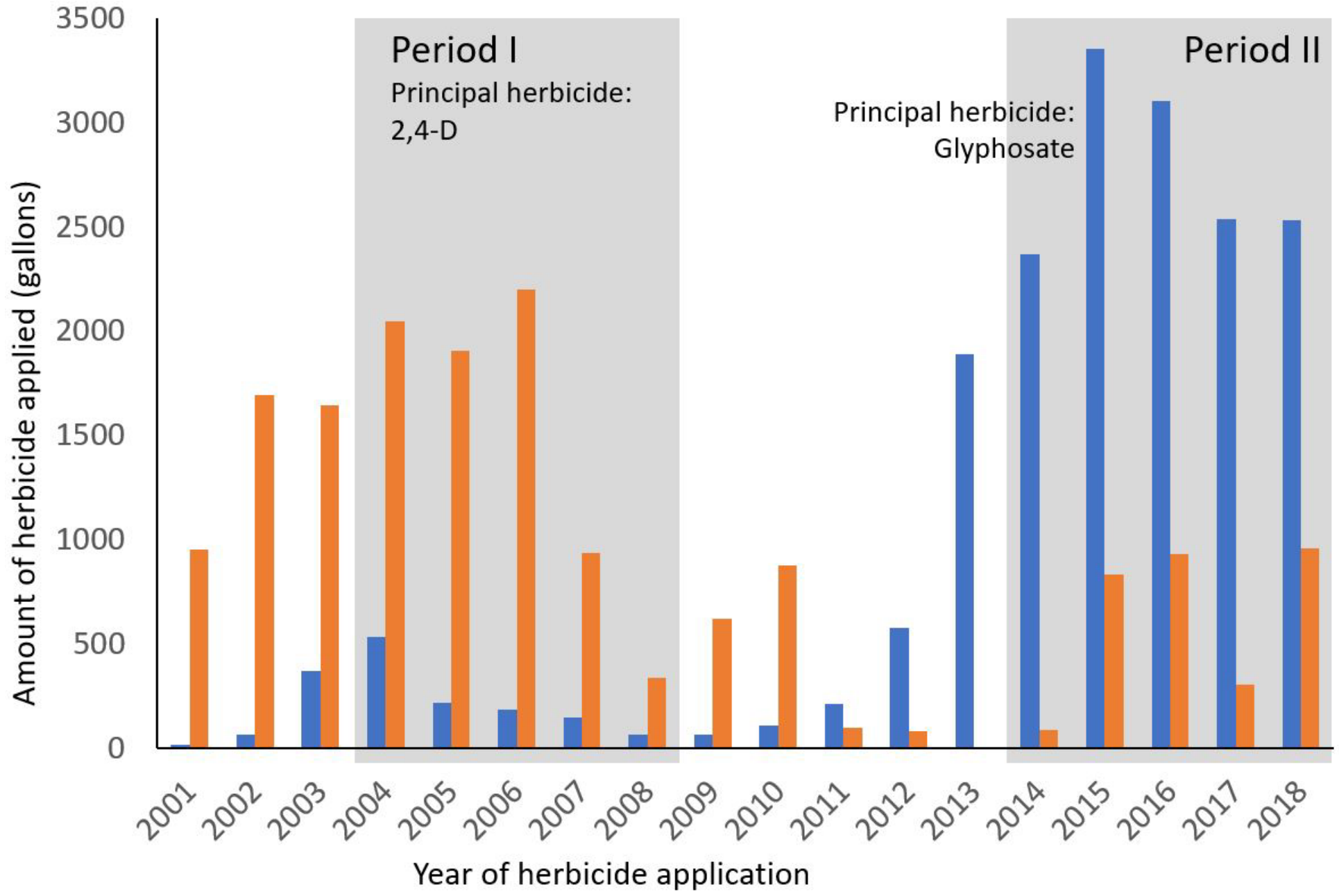

Figure 1. Amount of 2,4-D and glyphosate in gallons applied across the entire delta annually. Data derived from publicly available annual reports published by Division of Boating and Waterways (DBW). Gray region shows the years for which image data were available. The principal herbicide used during 2004-2008 was 2,4-D (orange bars), while glyphosate (blue bars) was used primarily in the 2014-2018 time period.

These log tables were used to compute the following information for each site treated in any year during the study period:

1. Herbicide type: 2,4-D or glyphosate
2. Application rate per unit area for the entire treatment season
3. Frequency of spraying within a treatment season (sprFreq)
4. Consecutive years of treatment until the present year (CY)

Habitat

The Delta is a diverse network of channels emptying the two major river systems of Northern and Central California into San Francisco Bay. While the Delta supports a great variety of habitats, it can be broadly characterized by three hydrologically distinct habitats as described below and illustrated in Figure 2:

Channels: Principal water channels in the Delta are wide and deep with rip-rap levees and do not support aquatic vegetation except along the shallow channel edges where water velocity is low. Channels with small remnant marsh islands support FAV in the shallow waters along the island's edge. Channel habitat is generally the most tidally open, with relatively higher water speeds and low water residence time.

Flooded Islands: When the Delta was reclaimed and channelized, it became a network of channels enclosing cultivated 'land' islands. Agricultural use on the islands required pumping water out to prevent water logging leading to subsidence. As a result, most of these islands today have subsided several feet and are below the water level in the channels. Over the decades, some of these islands had breached levees that were never repaired and allowed to become a part of the tidal habitat again. These flooded 'islands' surrounded by remnant levees often have shallow water depths and support extensive patches

of submerged and floating vegetation. Emergent wetland species like tules and cattails form a boundary at the leading edge of the land. Riparian vegetation on the levee protects the islands and acts as a buffer to the strong winds common in the Delta. These Deltaic lakes are characterized by low water velocity driven primarily by wind-wave action, low tidal action, and high water residence time.

Slow shallows: Within some major channels such as the Old River and Middle River in the Delta, the middle section of the channels have a series of remnant marshes. The waterways between these marshes are tidally open (low water residence time) but shallow with lower water speeds compared to the main channels. We call these regions, slow shallows. Similarly, there are many narrow and shallow meandering sloughs in the east Delta that do not have throughflow and just end at the edge of the legal delta limits. These dead-end sloughs are also hydrologically similar to slow shallows and extensively colonized by submerged and floating vegetation.

Once the Delta was divided into these 3 kinds of hydrologic habitats, we intersected this habitat layer with the study sites and assigned a habitat type to each site (Figure 2).

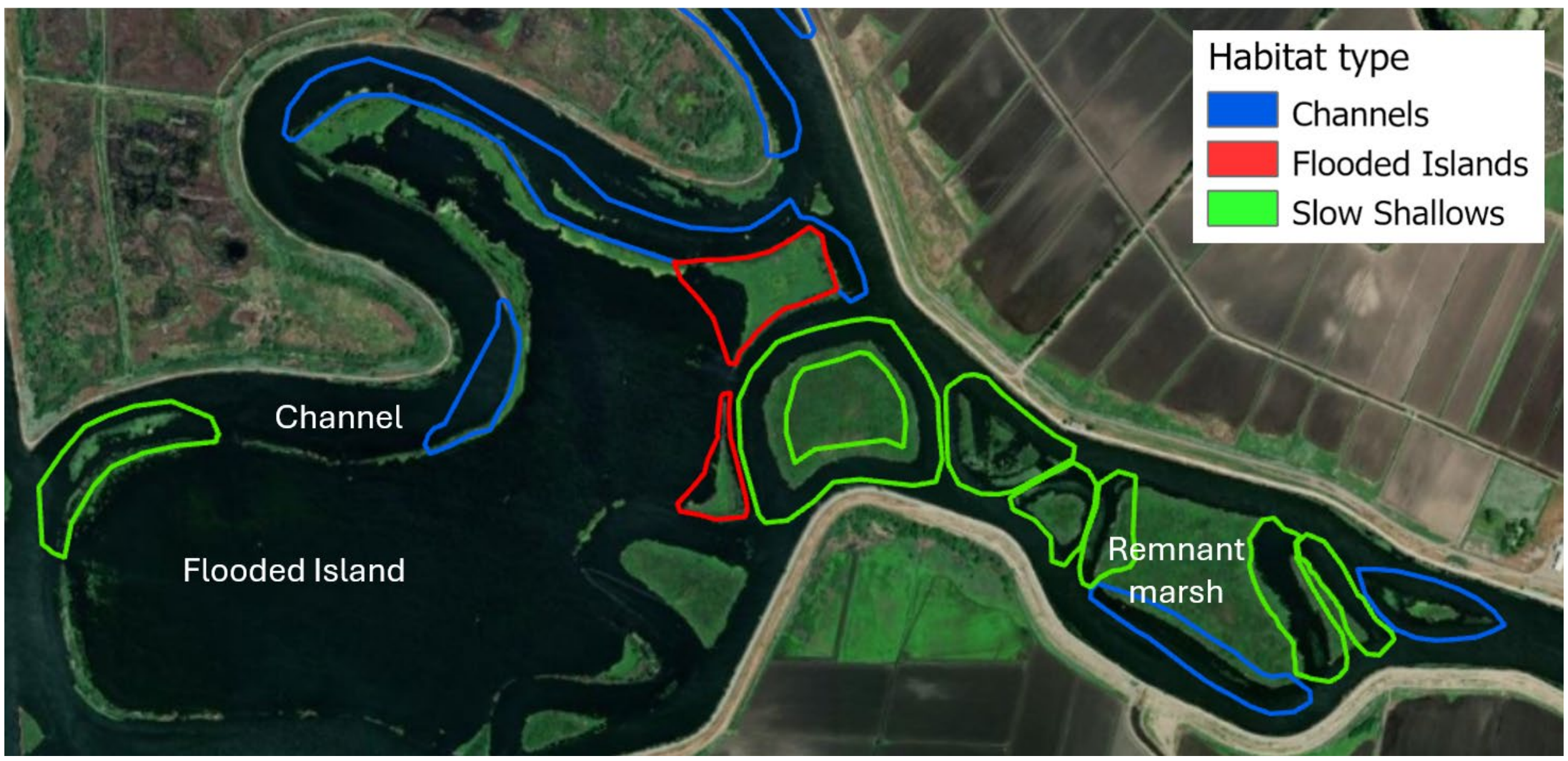

Figure 2. Channels, flooded islands, and remnant marshes in the Delta. Study sites were accordingly categorized as channel habitat, flooded island or slow shallows habitat.

Table 2 shows the number of samples (sites) available for each year and each CY.

Table 2: Total number of sites in each year for each value of continuous years of treatment (CY). All sites with CY = 0 are reference sites for that year.

**Year**	**Continuous years of treatment**							
	**0**	**1**	**2**	**3**	**4**	**5**	**6**	**> 6**
2004	151	22	3	177	0	0	0	0
2005	112	65	16	3	157	0	0	0
2006	94	41	54	14	1	149	0	0
2007	92	34	33	42	14	1	137	0
2008	101	35	28	25	33	13	1	115
2014	177	71	38	25	1	9	1	28
2015	101	95	59	33	23	1	9	29
2016	142	12	39	23	14	10	1	21
2017	141	41	6	21	18	9	5	22
2018	117	69	29	4	18	18	11	23

Results

For exploring the impact of treatment within the year of application of herbicide, a separate model was fit for each habitat for each time period (2004-2008 & 2014-2018) resulting in a total of six models for exploring the impact of treatment within the year of application.

For exploring the legacy effects of treatment, 1, 2, or 3 years after treatment, there were two suites of models. The first explored the legacy effect of treatment one year after herbicide application taking into account the history of treatment at the site (CY). This resulted in six models, one for each time period and habitat. The second explored the legacy effect of treatment taking into account the number of years that had passed since the last time the site was treated and not accounting for site treatment history. This resulted in another six models, one for each time period and habitat.

Coefficients, z-values and model validation plots for each of these 18 models are presented below.

Treatment impact within the year of application

Table 3. In channel habitat, herbicide efficacy mixed effects logistic (binomial) model results of the probability of FAV presence as a function of 2,4-D application, spray frequency (sprFreq), and consecutive treatment years binned in 2 year bins (CY2yrs) given 844 observations from 180 sites across 5 years (2004-2008). Parameters and coefficients correspond to Eq. S1.

**Random effects**	**Group**	**Parameter**	**Parameter variance**	**Sample Size**
	Site (intercept)	$\sigma^2_{\alpha_j}$	2.823	180
	Year (intercept)	$\sigma^2_{\alpha_k}$	0.233	5
**Fixed effects**	**Variable**	**Coefficient**	**Coefficient estimate (standard error)**	**z-value**
	Intercept	β_0	-3.319 (0.250)	-13.3
	$log_{10}herbicide$	β_1	-1.901 (0.012)	-164.82
	$\sqrt[3]{sprFreq}$	β_2	0.718 (0.010)	69.99
	CY2yrs1	β_3	-0.056 (0.019)	-2.92
	CY2yrs2	β_4	-0.076 (0.018)	-4.12
	CY2yrs3	β_5	0.059 (0.022)	2.64
	CY2yrs4	β_6	0.339 (0.024)	14.18

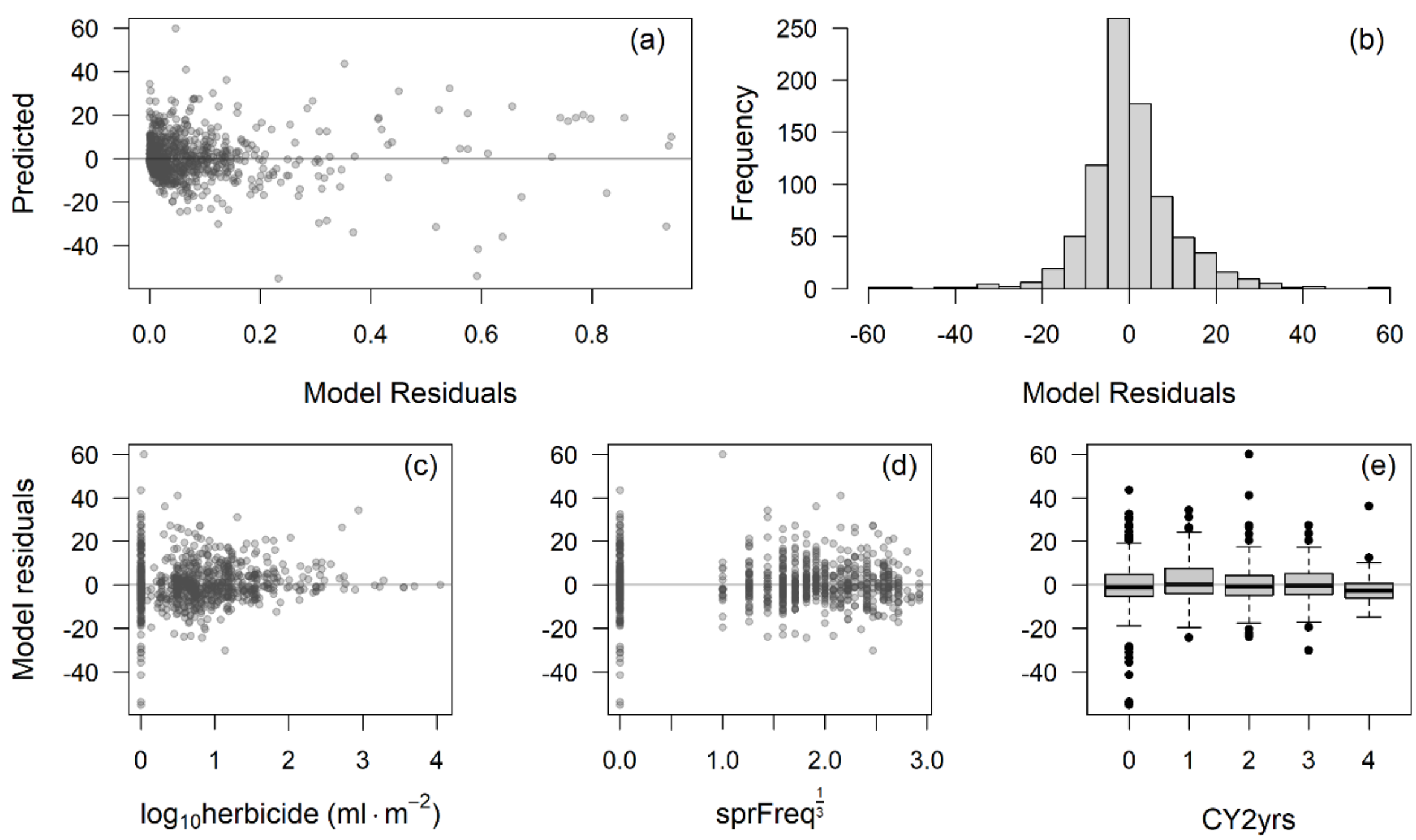

Figure 3: For channel habitat, treatment effect model fit as assessed via (a) residuals across predicted value, (b) a histogram of model residuals, and model residuals across the predictor variables of (c) herbicide (2,4-D), (d) spray frequency (sprFreq), and (e) consecutive treatment years binned in 2 year bins (CY2yrs) for the time period 2004-2008.

Table 4. In Slow Shallows habitat, herbicide efficacy mixed effects logistic (binomial) model results of the probability of FAV presence as a function of 2,4-D herbicide application, spray frequency (sprFreq), and consecutive treatment years binned in 2 year bins (CY2yrs), given 447 observations from 99 sites across 5 years (2004-2008). Parameters and coefficients correspond to Eq. S1.

Random effects	Group	Parameter	Parameter variance	Sample Size
	Site (intercept)	$\sigma^2_{\alpha_j}$	3.647	99
	Year (intercept)	$\sigma^2_{\alpha_k}$	0.462	5
**Fixed effects**	**Variable**	**Coefficient**	**Coefficient estimate (standard error)**	**z-value**
	Intercept	β_0	-2.226 (0.360)	-6.19
	$log_{10}herbicide$	β_1	-1.633 (0.016)	-99.86
	$\sqrt[3]{sprFreq}$	β_2	0.255 (0.014)	18.63
	CY2yrs1	β_3	0.615 (0.029)	21.3
	CY2yrs2	β_4	-0.318 (0.029)	-10.91
	CY2yrs4	β_5	0.188 (0.034)	5.57

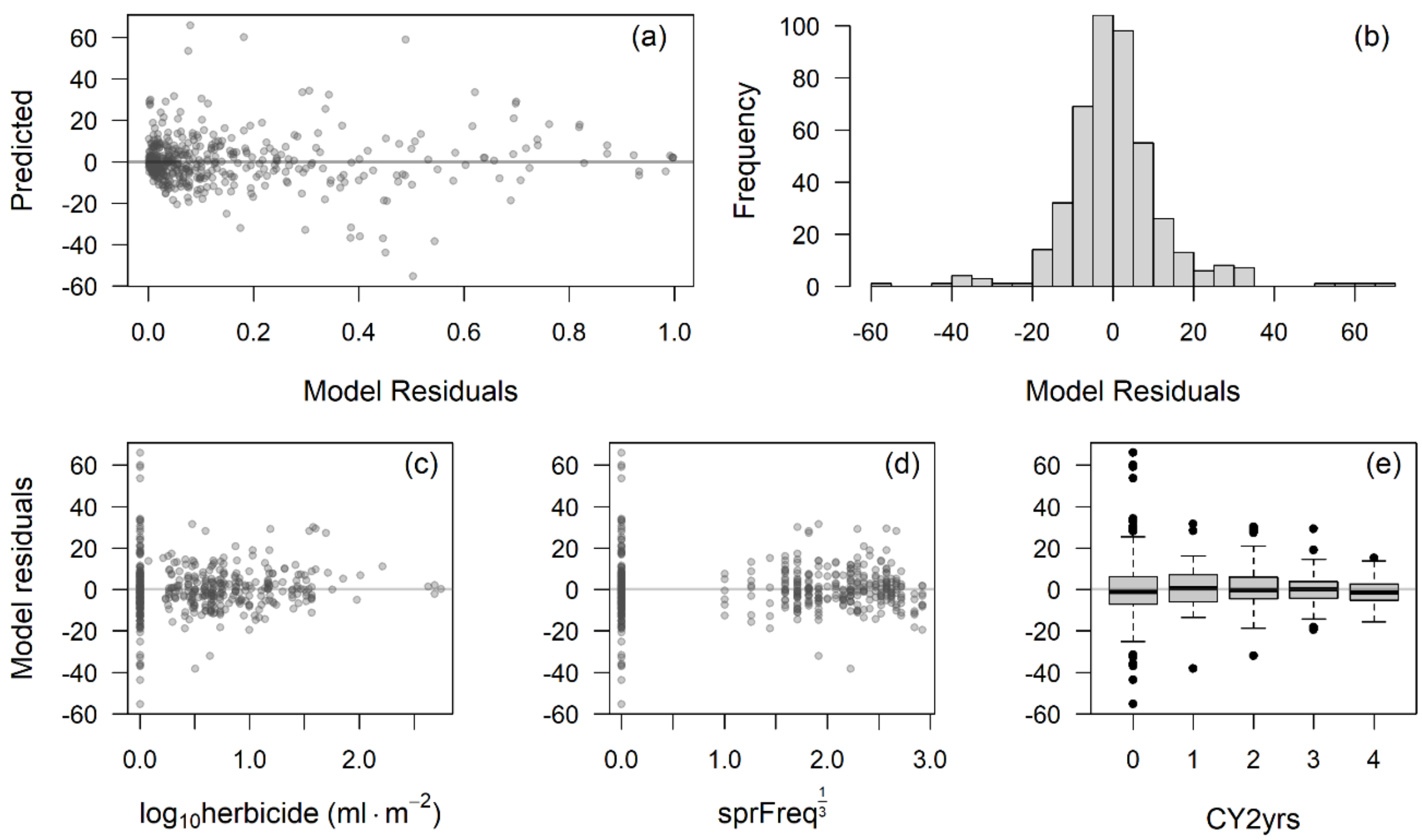

Figure 4: For Slow Shallows habitat, treatment effect model fit as assessed via (a) residuals across predicted value, (b) a histogram of model residuals, and model residuals across the predictor variables of (c) herbicide (2,4-D), (d) spray frequency (sprFreq), and (e) consecutive years of treatment binned in 2 year bins (CY2yrs) for the time period 2004-2008.

Table 5. In Flooded Island habitat, herbicide efficacy mixed effects logistic (binomial) model results of the probability of FAV presence as a function of 2,4-D application, spray frequency (sprFreq), and consecutive treatment years binned in 2 year bins (CY2yrs), given 343 observations from 70 sites across 5 years (2004-2008). Parameters and coefficients correspond to Eq. S1.

**Random effects**	**Group**	**Parameter**	**Parameter variance**	**Sample Size**
	Site (intercept)	$\sigma^2_{\alpha_j}$	2.759	70
	Year (intercept)	$\sigma^2_{\alpha_k}$	0.013	5
**Fixed effects**	**Variable**	**Coefficient**	**Coefficient estimate (standard error)**	**z-value**
	Intercept	β_0	-2.403 (0.205)	-11.71
	$log_{10}herbicide$	β_1	-1.322 (0.015)	-88.63
	$\sqrt[3]{sprFreq}$	β_2	0.050 (0.008)	6.11
	CY2yrs1	β_3	0.233 (0.017)	13.57
	CY2yrs2	β_4	0.186 (0.017)	10.69
	CY2yrs3	β_5	-0.037 (0.020)	-1.87
	CY2yrs4	β_6	-0.025 (0.022)	-1.14

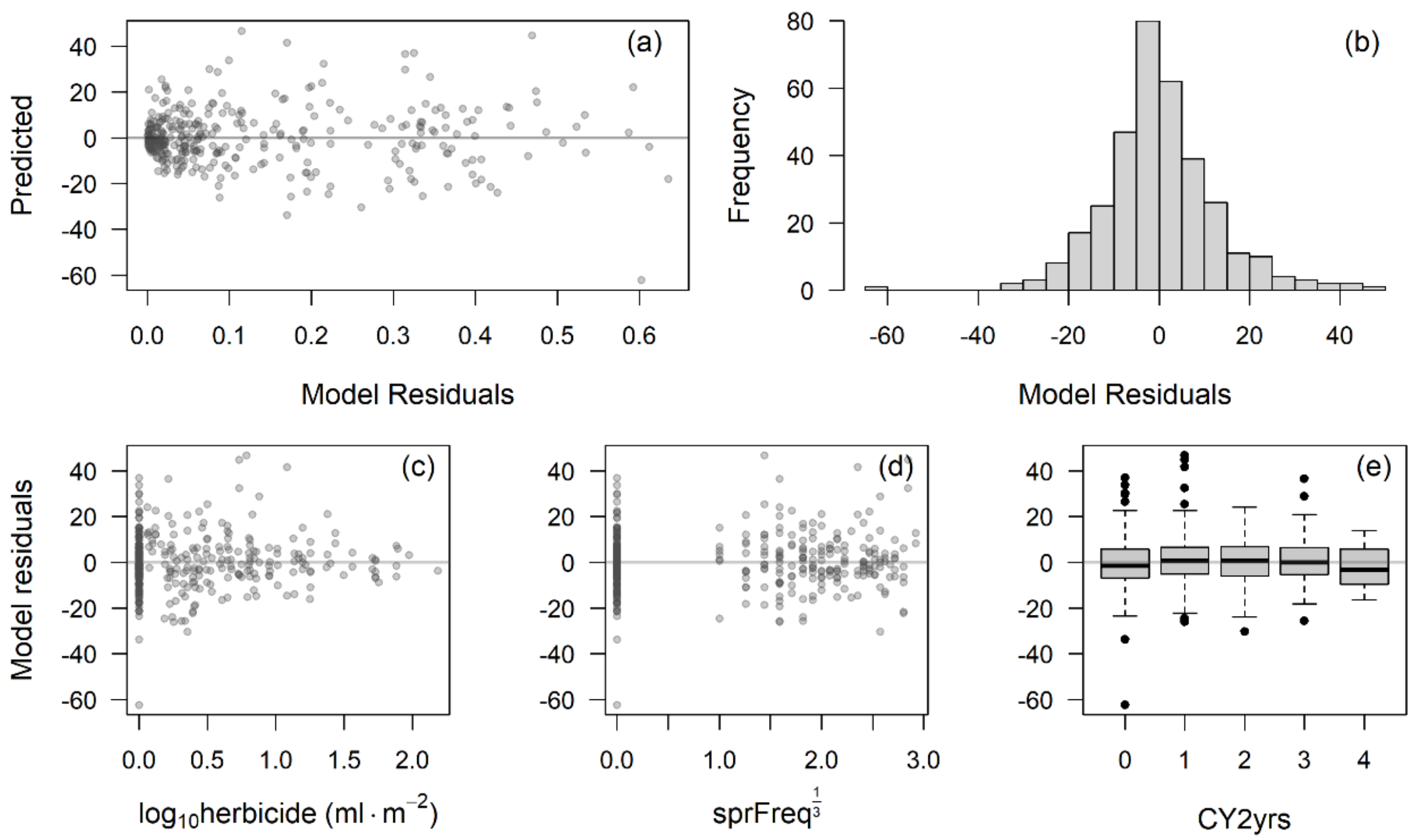

Figure 5: For Flooded Islands habitat, treatment effect model fit as assessed via (a) residuals across predicted value, (b) a histogram of model residuals, and model residuals across the predictor variables of (c) herbicide (2,4-D), (d) spray frequency (sprFreq), and (e) consecutive years of treatment binned in 2 year bins (CY2yrs) for the time period 2004-2008.

Table 6. In Channel habitat, herbicide efficacy mixed effects logistic (binomial) model results of the probability of FAV presence as a function of glyphosate application, spray frequency (sprFreq), and consecutive treatment years binned in 2 year bins (CY2yrs) given 495 observations from 167 sites across 5 years (2014-2018). Parameters and coefficients correspond to Eq. S1.

Random effects	Group	Parameter	Parameter variance	Sample Size
	Site (intercept)	$\sigma^2_{\alpha_j}$	1.454	167
	Year (intercept)	$\sigma^2_{\alpha_k}$	0.258	5
**Fixed effects**	**Variable**	**Coefficient**	**Coefficient estimate (standard error)**	**z-value**
	Intercept	β_0	-1.504 (0.246)	-6.12
	$log_{10}herbicide$	β_1	-1.431 (0.010)	-143.97
	$\sqrt[3]{sprFreq}$	β_2	0.632 (0.010)	64.87
	CY2yrs1	β_3	-0.483 (0.014)	-34.90
	CY2yrs2	β_4	-0.415 (0.015)	-28.32
	CY2yrs3	β_5	-0.338 (0.017)	-20.14
	CY2yrs4	β_6	1.232 (0.033)	37.68

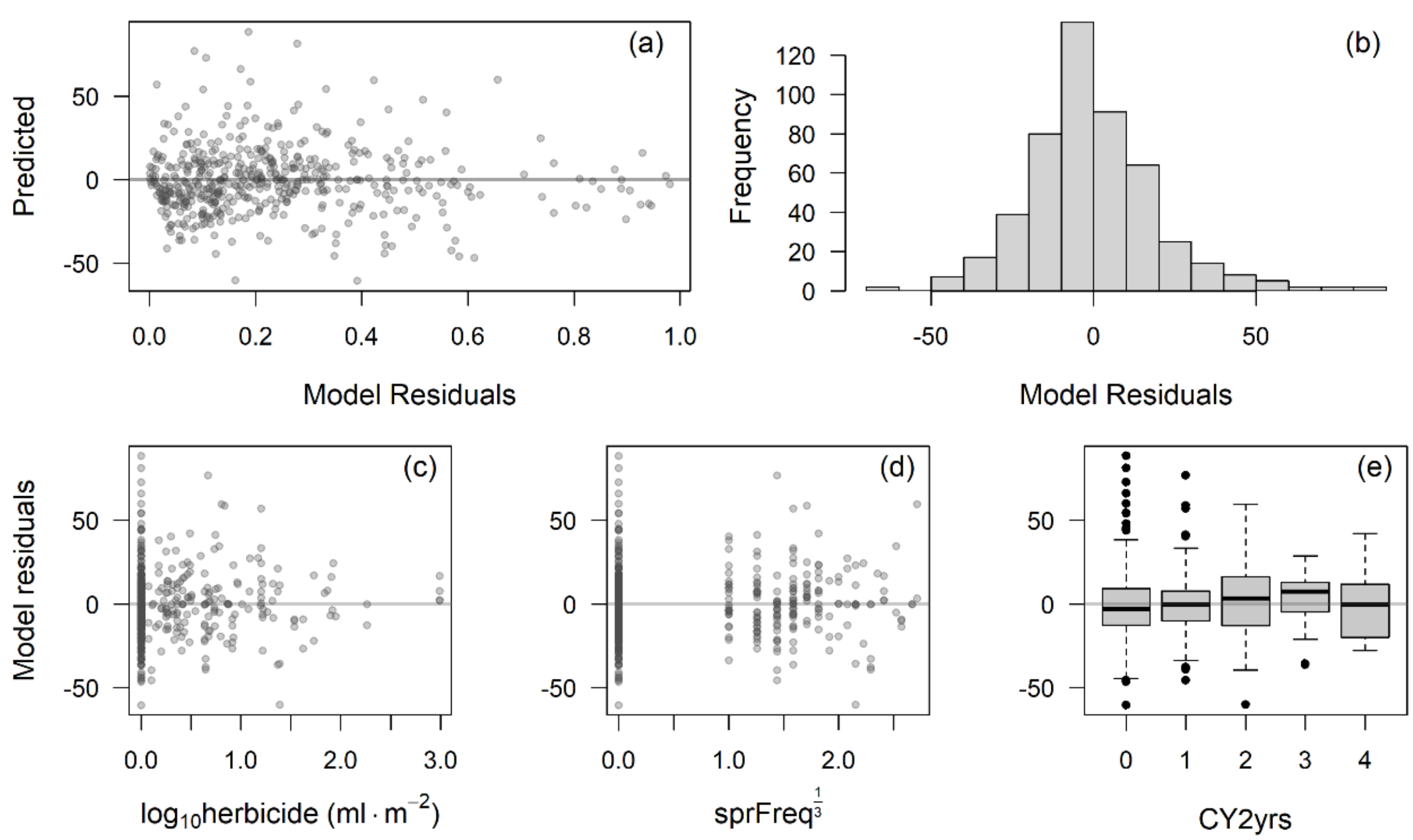

Figure 6: For channel habitat, treatment effect model fit as assessed via (a) residuals across predicted value, (b) a histogram of model residuals, and model residuals across the predictor variables of (c) herbicide (glyphosate), (d) spray frequency (sprFreq), and (e) consecutive treatment years binned in 2 year bins (CY2yrs) for the time period 2014-2018.

Table 7. In Slow Shallows habitat, herbicide efficacy mixed effects logistic (binomial) model results of the probability of FAV presence as a function of glyphosate herbicide application, spray frequency (sprFreq), and consecutive treatment years binned in 2 year bins (CY2yrs), given 272 observations from 98 sites across 5 years (2014-2018). Parameters and coefficients correspond to Eq. S1.

Random effects	Group	Parameter	Parameter variance	Sample Size
	Site (intercept)	$\sigma^2_{\alpha_j}$	3.518	98
	Year (intercept)	$\sigma^2_{\alpha_k}$	0.083	5
**Fixed effects**	**Variable**	**Coefficient**	**Coefficient estimate (standard error)**	**z-value**
	Intercept	β_0	-0.721 (0.229)	-3.15
	$log_{10}herbicide$	β_1	-3.022 (0.015)	-200.24
	$\sqrt[3]{sprFreq}$	β_2	1.856 (0.013)	147.58
	CY2yrs1	β_3	-1.385 (0.019)	-73.01
	CY2yrs2	β_4	-1.181 (0.018)	-65.05
	CY2yrs4	β_5	-1.082 (0.023)	-46.55

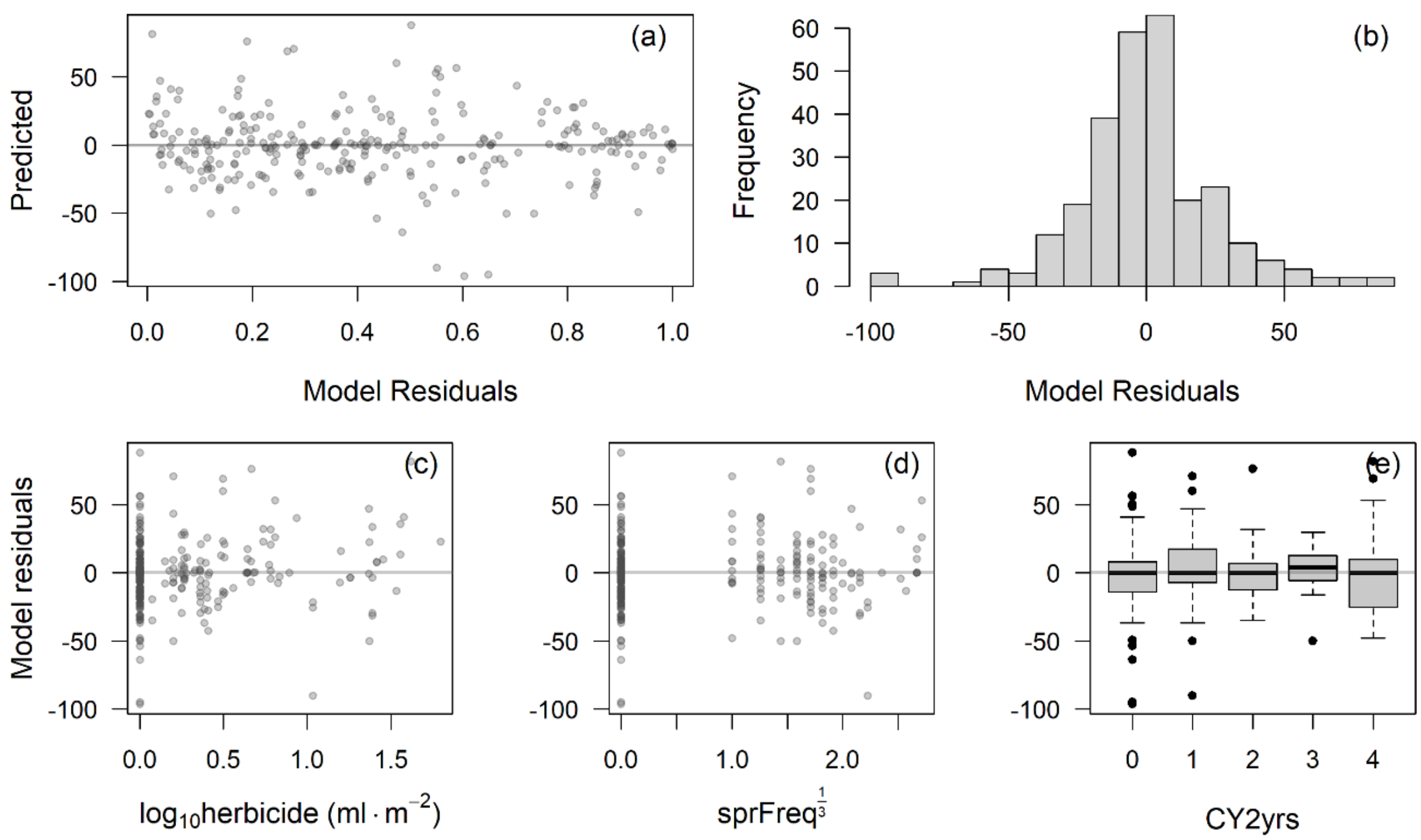

Figure 7: For Slow Shallows habitat, treatment effect model fit as assessed via (a) residuals across predicted value, (b) a histogram of model residuals, and model residuals across the predictor variables of (c) herbicide (glyphosate), (d) spray frequency (sprFreq), and (e) consecutive years of treatment binned in 2 year bins (CY2yrs) for the time period 2014-2018.

Table 8. In Flooded Island habitat, herbicide efficacy mixed effects logistic (binomial) model results of the probability of FAV presence as a function of glyphosate application, spray frequency (sprFreq), and consecutive treatment years binned in 2 year bins (CY2yrs), given 262 observations from 68 sites across 5 years (2014-2018). Parameters and coefficients correspond to Eq. S1.

Random effects	Group	Parameter	Parameter variance	Sample Size
	Site (intercept)	$\sigma^2_{\alpha_j}$	1.450	68
	Year (intercept)	$\sigma^2_{\alpha_k}$	0.032	5
**Fixed effects**	**Variable**	**Coefficient**	**Coefficient estimate (standard error)**	**z-value**
	Intercept	β_0	-0.375 (0.166)	-2.25
	$log_{10}herbicide$	β_1	-2.185 (0.020)	-108.18
	$\sqrt[3]{sprFreq}$	β_2	1.828 (0.018)	102.24
	CY2yrs1	β_3	-1.613 (0.020)	-78.9
	CY2yrs2	β_4	-2.120 (0.026)	-82.06
	CY2yrs3	β_5	-2.357 (0.048)	-49.18
	CY2yrs4	β_6	-0.375 (0.166)	-2.25

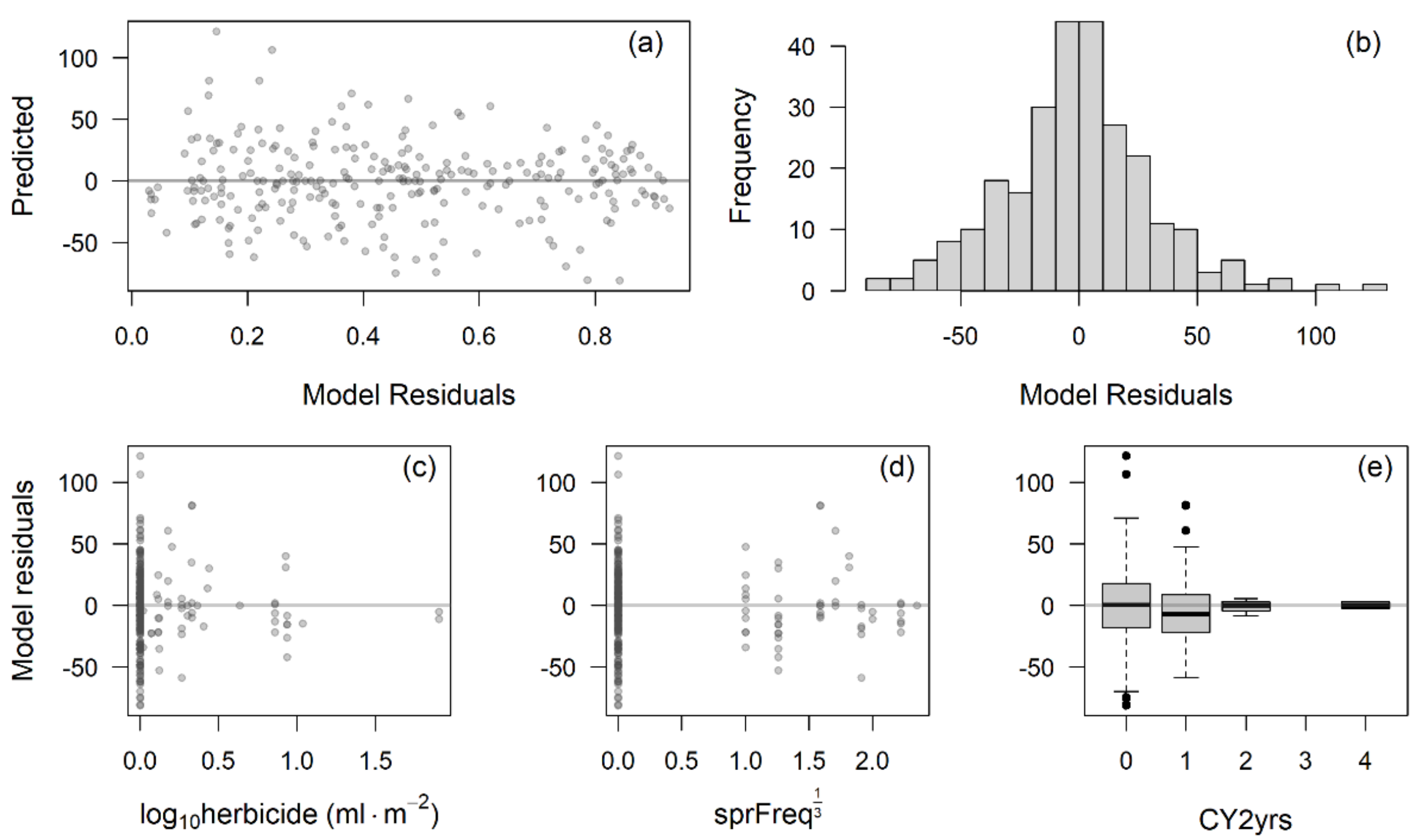

Figure 8: For Flooded Islands habitat, treatment effect model fit as assessed via (a) residuals across predicted value, (b) a histogram of model residuals, and model residuals across the predictor variables of (c) herbicide (glyphosate), (d) spray frequency (sprFreq), and (e) consecutive years of treatment binned in 2 year bins (CY2yrs) for the time period 2014-2018.

Legacy effect of treatment

Table 9. Herbicide legacy effects mixed effects logistic (binomial) model results for the time period 2004-2008 of the probability of FAV presence in reference sites during time *t* as a function of consecutive treatment years during the previous year (CY_{t-1}; a categorical variable). Total observations in channel habitat were 190, in flooded islands were 127, and in slow shallows were 134. Parameters and coefficients correspond to Eq. S2.

**Random effects**	**Habitat**	**Group**	**Parameter**	**Parameter variance**	**Sample Size**
	Channel	Site (intercept)	$\sigma^2_{\alpha_j}$	7.643	110
		Year (intercept)	$\sigma^2_{\alpha_k}$	1.302	5
	Slow Shallows	Site (intercept)	$\sigma^2_{\alpha_j}$	10.373	62
		Year (intercept)	$\sigma^2_{\alpha_k}$	0.687	5
	Flooded Island	Site (intercept)	$\sigma^2_{\alpha_j}$	3.108	52
		Year (intercept)	$\sigma^2_{\alpha_k}$	0.006	5
**Fixed effects**	**Habitat**	**Variable**	**Coefficient**	**Coefficient estimate (standard error)**	**z-value**
	Channel	Intercept	β_0	-5.113 (0.587)	-8.71
		$CY2yrs_{t-1}1$	β_1	1.154 (0.039)	29.52
		$CY2yrs_{t-1}2$	β_2	1.016 (0.054)	18.94
		$CY2yrs_{t-1}3$	β_3	1.527 (0.754)	2.03
	Slow Shallows	Intercept	β_0	-3.730 (0.608)	-6.13
		$CY2yrs_{t-1}1$	β_1	1.521 (0.043)	35.35
		$CY2yrs_{t-1}2$	β_2	2.971 (0.779)	3.82
		$CY2yrs_{t-1}3$	β_3	1.260 (0.966)	1.3
	Flooded Island	Intercept	β_0	-2.830 (0.255)	-11.09
		$CY2yrs_{t-1}1$	β_1	0.127 (0.032)	3.93
		$CY2yrs_{t-1}2$	β_2	0.128 (0.056)	2.29
		$CY2yrs_{t-1}3$	β_3	-0.016 (1.050)	-0.02

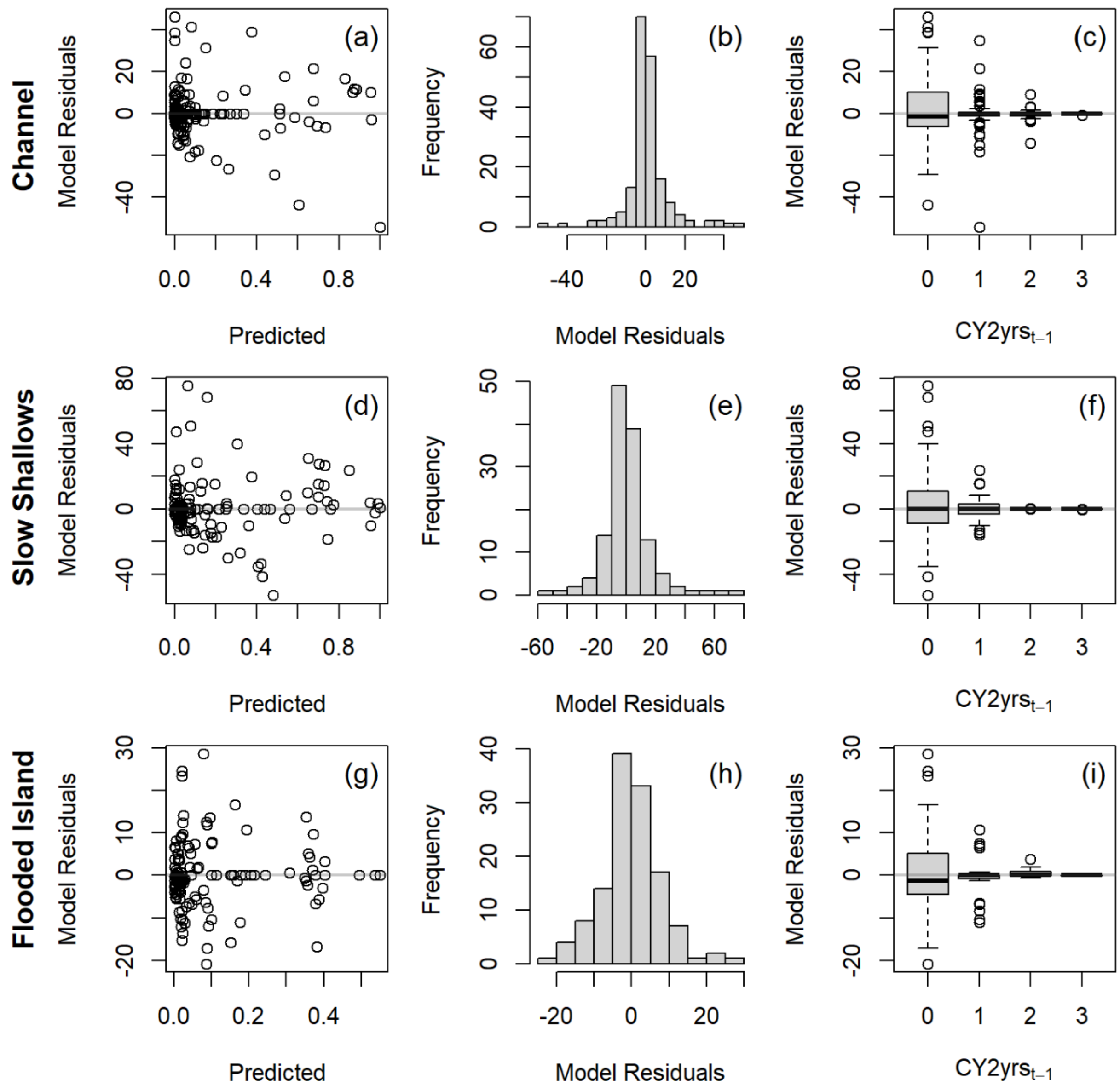

Figure 9: Legacy effect model fit for channel habitat for the years 2004-2008 as assessed via (a) model residuals across predicted value, (b) a histogram of model residuals, and model residuals across (c) consecutive treatment years as of the previous year binned in 2 year intervals (CY2yrs$_{t-1}$). Similarly, model fit for flooded islands (d, e, f) and slow shallows (g, h, i).

Table 10. Herbicide legacy effects mixed effects logistic (binomial) model results for the time period 2004-2008 of the probability of FAV presence in reference sites as a function of "years since last treated" (T_{flag}; a categorical variable). Total observations in channel habitat were 231, in flooded islands were 153, and in slow shallows were 166. Parameters and coefficients correspond to Eq. S2.

Random effects	Habitat	Group	Parameter	Parameter variance	Sample Size
	Channel	Site (intercept)	$\sigma^2_{\alpha_j}$	6.114	110
		Year (intercept)	$\sigma^2_{\alpha_k}$	0.927	5
	Slow Shallows	Site (intercept)	$\sigma^2_{\alpha_j}$	9.724	62
		Year (intercept)	$\sigma^2_{\alpha_k}$	0.576	5
	Flooded Island	Site (intercept)	$\sigma^2_{\alpha_j}$	3.146	52
		Year (intercept)	$\sigma^2_{\alpha_k}$	0.007	5
**Fixed effects**	**Habitat**	**Variable**	**Coefficient**	**Coefficient estimate (standard error)**	**z-value**
	Channel	Intercept	β_0	-4.445 (0.492)	-9.03
		$T_{flag} = 1$	β_1	0.573 (0.032)	17.93
		$T_{flag} = 2$	β_2	0.112 (0.035)	3.24
		$T_{flag} = 3$	β_3	-0.725 (0.044)	-16.54
	Slow Shallows	Intercept	β_0	-3.090 (0.524)	-5.9
		$T_{flag} = 1$	β_1	0.856 (0.036)	23.53
		$T_{flag} = 2$	β_2	0.299 (0.041)	7.26
		$T_{flag} = 3$	β_3	-0.520 (0.041)	-12.59
	Flooded Island	Intercept	β_0	-2.838 (0.250)	-11.37
		$T_{flag} = 1$	β_1	0.059 (0.029)	2.04
		$T_{flag} = 2$	β_2	0.151 (0.030)	4.97
		$T_{flag} = 3$	β_3	0.219 (0.031)	6.99

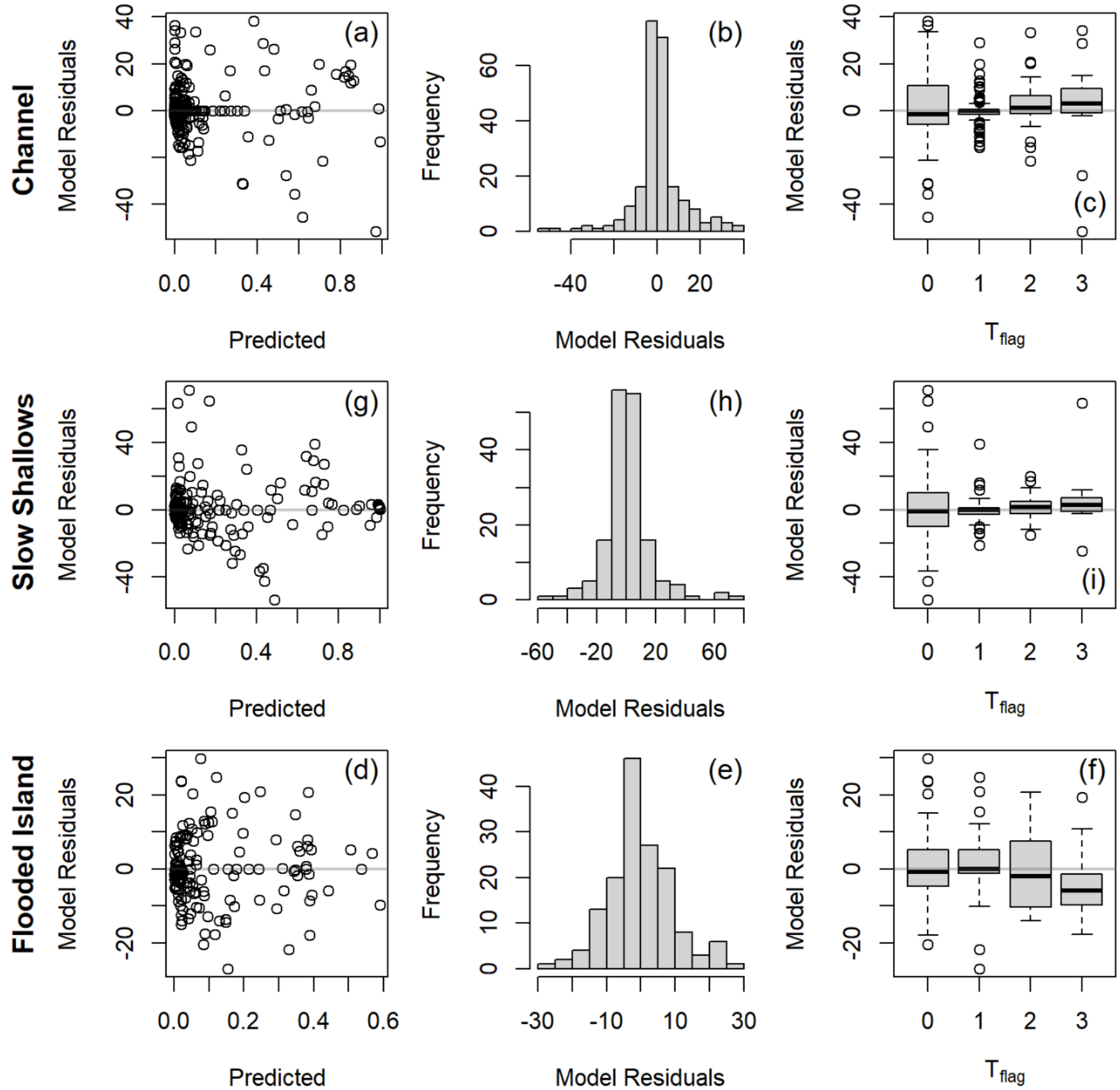

Figure 10. Legacy effect model fit for years 2004-2008 as assessed via (a) model residuals across predicted value, (b) a histogram of model residuals, and model residuals across (c) consecutive treatment years as of the previous year binned in 2 year intervals ($CY2yrs_{t-1}$). Similarly, model fit for flooded islands (d, e, and f) and slow shallows (g, h, and i).

Table 11. Herbicide legacy effects mixed effects logistic (binomial) model results for the years 2014-2018 of the probability of FAV presence in reference sites during time *t* as a function of consecutive treatment years during the previous year ($CY2yrs_{t-1}$; a categorical variable binned in 2 year increments). Total observations in channel habitat were 227, in flooded islands were 164, and in slow shallows were 113. Parameters and coefficients correspond to Eq. S2.

Random effects	Habitat	Group	Parameter	Parameter variance	Sample Size
	Channel	Site (intercept)	$\sigma^2_{\alpha_j}$	3.549	127
		Year (intercept)	$\sigma^2_{\alpha_k}$	0.226	5
	Slow Shallows	Site (intercept)	$\sigma^2_{\alpha_j}$	4.906	63
		Year (intercept)	$\sigma^2_{\alpha_k}$	0.885	5
	Flooded Island	Site (intercept)	$\sigma^2_{\alpha_j}$	1.171	60
		Year (intercept)	$\sigma^2_{\alpha_k}$	0.885	5
**Fixed effects**	**Habitat**	**Variable**	**Coefficient**	**Coefficient estimate (standard error)**	**z-value**
	Channel	Intercept	β_0	-1.958 (0.271)	-7.24
		$CY2yrs_{t-1}1$	β_1	-0.299 (0.010)	-31.36
		$CY2yrs_{t-1}2$	β_2	0.771 (0.019)	39.79
		$CY2yrs_{t-1}3$	β_3	1.075 (0.034)	32.05
	Slow Shallows	Intercept	β_0	1.117 (0.512)	2.18
		$CY2yrs_{t-1}1$	β_1	-2.188 (0.023)	-93.56
		$CY2yrs_{t-1}2$	β_2	-1.906 (0.043)	-44.71
		$CY2yrs_{t-1}3$	β_3	-7.653 (1.051)	-7.28
	Flooded Island	Intercept	β_0	0.146 (0.164)	0.89
		$CY2yrs_{t-1}1$	β_1	-0.645 (0.006)	-108.04

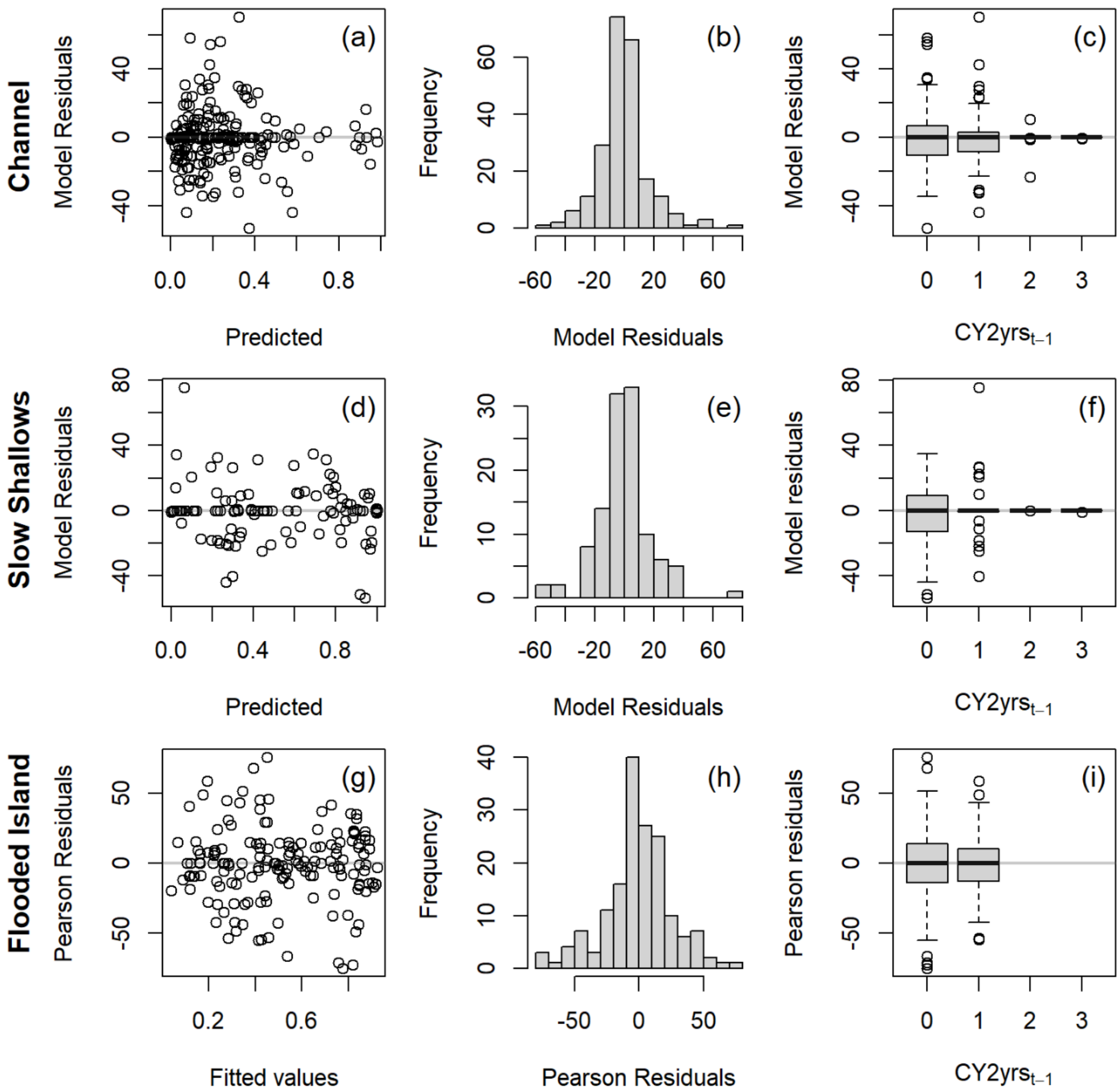

Figure 11: Legacy effect model fit for channel habitat for the years 2014-2018 as assessed via (a) model residuals across predicted value, (b) a histogram of model residuals, and model residuals across (c) consecutive treatment years as of the previous year binned in 2 year intervals (CY2yrs$_{t-1}$). Similarly, model fit for flooded islands (d, e, f) and slow shallows (g, h, i).

Table 12. Herbicide legacy effects mixed effects logistic (binomial) model results for the years 2014-2018 of the probability of FAV presence in reference sites as a function of "years since last treated" (T_{flag}; a categorical variable). Total observations in channel habitat were 315, in flooded islands were 210, and in slow shallows were 153. Parameters and coefficients correspond to Eq. S2.

**Random effects**	**Habitat**	**Group**	**Parameter**	**Parameter variance**	**Sample Size**
	Channel	Site (intercept)	$\sigma^2_{\alpha_j}$	2.139	129
		Year (intercept)	$\sigma^2_{\alpha_k}$	0.238	5
	Slow Shallows	Site (intercept)	$\sigma^2_{\alpha_j}$	6.251	67
		Year (intercept)	$\sigma^2_{\alpha_k}$	0.133	5
	Flooded Island	Site (intercept)	$\sigma^2_{\alpha_j}$	1.325	62
		Year (intercept)	$\sigma^2_{\alpha_k}$	0.021	5
**Fixed effects**	**Habitat**	**Variable**	**Coefficient**	**Coefficient estimate (standard error)**	**z-value**
	Channel	Intercept	β_0	-1.644 (0.254)	-6.48
		$T_{flag} = 1$	β_1	-0.146 (0.007)	-20.53
		$T_{flag} = 2$	β_2	-0.160 (0.007)	-22.08
		$T_{flag} = 3$	β_3	-0.250 (0.008)	-32.05
	Slow Shallows	Intercept	β_0	-0.228 (0.347)	-0.66
		$T_{flag} = 1$	β_1	-0.447 (0.014)	-32.65
		$T_{flag} = 2$	β_2	-0.477 (0.014)	-34.74
		$T_{flag} = 3$	β_3	-0.488 (0.016)	-31.03
	Flooded Island	Intercept	β_0	0.186 (0.160)	1.16
		$T_{flag} = 1$	β_1	-0.730 (0.005)	-135.47
		$T_{flag} = 2$	β_2	-1.246 (0.007)	-186.32
		$T_{flag} = 3$	β_3	-0.646 (0.007)	-98.39

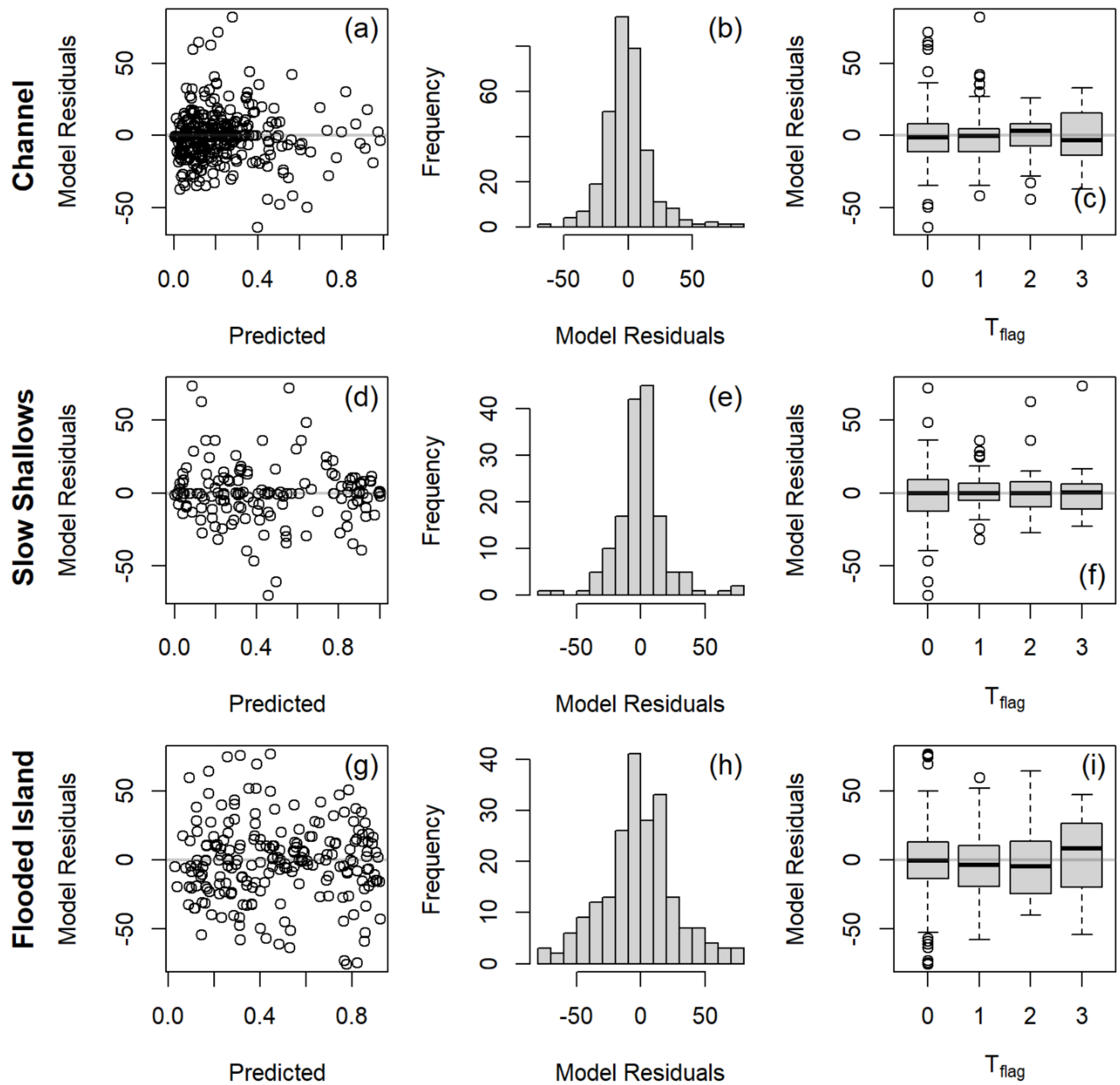

Figure 12. Legacy effect model fit for the time period 2014-2018 as assessed via (a) model residuals across predicted value, (b) a histogram of model residuals, and model residuals across (c) consecutive treatment years as of the previous year binned in 2 year intervals ($CY2yrs_{t-1}$). Similarly, model fit for flooded islands (d, e, and f) and slow shallows (g, h, and i).
